# Supplementary material for: Further development and feasibility randomised controlled trial of a digital programme for adolescent depression, MoodHwb: study protocol
Source: BMJ Open. 2023 Jun 5;13(6):e070369. doi: 10.1136/bmjopen-2022-070369 (PMC10254867; doi:10.1136/bmjopen-2022-070369)
Supplement: Author's manuscript [file bmjopen-2022-070369.draft_revisions.pdf]

BMJ Open is committed to open peer review. As part of this commitment we make the peer review history of every article we publish publicly available.

When an article is published we post the peer reviewers' comments and the authors' responses online. We also post the versions of the paper that were used during peer review. These are the versions that the peer review comments apply to.

The versions of the paper that follow are the versions that were submitted during the peer review process. They are not the versions of record or the final published versions. They should not be cited or distributed as the published version of this manuscript.

BMJ Open is an open access journal and the full, final, typeset and author-corrected version of record of the manuscript is available on our site with no access controls, subscription charges or pay-per-view fees (http://bmjopen.bmj.com).

If you have any questions on BMJ Open's open peer review process please email info.bmjopen@bmj.com

BMJ Open

Further development and feasibility randomised controlled trial of a digital programme for adolescent depression, MoodHwb: study protocol

Journal:	*BMJ Open*
Manuscript ID	bmjopen-2022-070369
Article Type:	Protocol
Date Submitted by the Author:	26-Nov-2022
Complete List of Authors:	Bevan Jones, R; Cardiff University, Division of Psychological Medicine and Clinical Neurosciences; Cwm Taf Morgannwg University Health Board<br>Merry, S; The University of Auckland, Faculty of Medical and Health Sciences<br>Stallard, Paul; University of Bath, Department for Health<br>Randell, Elizabeth; Cardiff University, Centre for Trials Research<br>Weavers, Bryony; Cardiff University, Division of Psychological Medicine and Clinical Neurosciences<br>Gray, Anna; Cardiff University, Division of Psychological Medicine and Clinical Neurosciences<br>Hindle, Elaine; University of Glasgow, MRC/CSO Social and Public Health Sciences Unit<br>Gavigan, Marcela; University of Glasgow, MRC/CSO Social and Public Health Sciences Unit<br>Clarkstone, Samantha; Cardiff University<br>Williams-Thomas, Rhys; Cardiff University, Centre for Trials Research<br>Poile, Vince; Cardiff University, Centre for Trials Research<br>Playle, Rebecca; Cardiff University, Centre for Trials Research<br>Bisson, Jonathan ; Cardiff University, Division of Psychological Medicine and Clinical Neurosciences<br>McNamara, Rachel; Cardiff University, Centre for Trials Research<br>Rice, F; Cardiff University, Division of Psychological Medicine and Clinical Neurosciences<br>Simpson, Sharon; University of Glasgow, MRC/CSO Social and Public Health Sciences Unit
Keywords:	Child & adolescent psychiatry < PSYCHIATRY, MENTAL HEALTH, Depression & mood disorders < PSYCHIATRY, Clinical trials < THERAPEUTICS

SCHOLARONE™
Manuscripts

Further development and feasibility randomised controlled trial of a digital programme for adolescent depression, MoodHwb: study protocol

Rhys Bevan Jones[1-3], Sally Merry[4], Paul Stallard[5], Elizabeth Randell[6], Bryony Weavers[1,2], Anna Gray[1,2], Elaine Hindle[7], Marcela Gavigan[7], Samantha Clarkstone[6], Rhys Williams-Thomas[6], Vincent Poile[6], Rebecca Playle [6], Jonathan Bisson[1,2], Rachel McNamara[6], *Frances Rice[1,2], *Sharon Anne Simpson[7]

*Joint senior authors

1. Division of Psychological Medicine and Clinical Neurosciences, Cardiff University, Wales.
2. Wolfson Centre for Young People's Mental Health, Cardiff University, Wales.
3. Cwm Taf Morgannwg University Health Board, Wales.
4. Faculty of Medical and Health Sciences, University of Auckland, New Zealand.
5. Department for Health at the University of Bath, England.
6. Centre for Trials Research, Cardiff University, Wales.
7. Medical Research Council/Chief Scientist Office (MRC/CSO) Social and Public Health Sciences Unit, University of Glasgow, Scotland.

Corresponding author:

Dr Rhys Bevan Jones, Division of Psychological Medicine & Clinical Neurosciences, Cardiff University, Hadyn Ellis Building, Maindy Rd, Cardiff, Wales CF24 4HQ. Email: bevanjonesr1@cardiff.ac.uk

Word count 4000

For peer review only

Abstract

Introduction: A digital programme, MoodHwb, was co-designed with young people experiencing or at high risk of depression, parents/carers, and professionals, to provide support for young people with their mood and wellbeing. A preliminary evaluation study provided support for the programme theory and found that MoodHwb was acceptable to use. The present study aims to refine the programme based on user feedback, and to assess the acceptability and feasibility of the updated version and study methods.

Methods and analysis: Initially, this study will refine MoodHwb with the involvement of young people, including in a pre-trial acceptability phase. This will be followed by a multi-centre feasibility randomised controlled trial comparing MoodHwb plus usual care with a digital information pack plus usual care. Up to 120 young people aged 13-19 years with symptoms of depression and their parents/carers will be recruited through schools, primary mental health services, youth services, charities, and voluntary self-referral in Wales and Scotland. The primary outcomes are the feasibility and acceptability of the MoodHwb programme (including usage, design, and content) and of trial methods (including recruitment and retention rates), assessed two months post-randomisation. Secondary outcomes include potential impact on domains including depression knowledge and stigma, help-seeking, wellbeing and depression and anxiety symptoms measured at two months post-randomisation.

Ethics and dissemination: The pre-trial acceptability phase was approved by the Cardiff University School of Medicine Research Ethics Committee (REC) and the University of Glasgow College of Medicine, Veterinary and Life Sciences REC. The trial was approved by Wales NHS REC 3 (21/WA/0205), the Health Research Authority (HRA), Health and Care Research Wales (HCRW), university health board R&D departments in Wales, and schools in Wales and Scotland. Findings will be disseminated in peer-reviewed open-access journals, at conferences and meetings, and online to academic, clinical, and educational audiences and the wider public.

Trial registration number: ISRCTN12437531.

Strengths and limitations of this study

- MoodHwb was co-designed with young people with lived experience or at high risk of depression, parents/carers, and practitioners.

For peer review only

- MoodHwb is bilingual, personalised according to user needs and preferences, and has been developed in line with evidence-based approaches to support young people with depressive symptoms and their parents/carers, friends, and practitioners.
- Multiple methods will be used to collect data in a range of settings for the trial, including validated questionnaires, semi-structured interviews, a focus group, and Web/app usage monitoring.
- As this is a feasibility study, we will not be able to determine the effectiveness of MoodHwb; however, the findings will inform the design of a definitive effectiveness trial.
- Participants will not be blind to treatment allocation as this is not possible with an intervention of this kind.

For peer review only

BACKGROUND

Depression is common in adolescence, and is associated with social and educational impairments, deliberate self-harm, and suicide. It can also mark the beginning of long-term mental health difficulties. Subthreshold depressive symptoms can also affect quality of life and are a risk indicator for depressive disorder. Early treatment and prevention of adolescent depression is therefore a major public health concern[1]. There are also concerns about the economic burden of mental disorders[2,3]. However, most adolescents with depression do not access or receive formal help and engaging young people (YP) in prevention and early intervention programmes is challenging[4-6].

Guidelines for the prevention and management of depression in YP (e.g., NICE[7]; AACAP[8]) stress the need for good information and evidence-based psychosocial interventions for YP and families/carers, and digital interventions are recommended for mild depression in UK NICE guidelines[7]. Digital mental health technologies (i.e., resources and interventions) are a potential way of increasing access to support, at relatively low cost. Most YP in high-income countries have access to the internet and mobile devices, including in the UK[9,10]. Many in low and middle-income countries (LMICs) can also access the internet, where there are few alternative sources of support[11]. Whilst there is increasing evidence for the effectiveness of technologies for adolescent depression[12,13], more programmes are needed to support adolescents that are accessible, evidence-based, developed with user input and evaluated in line with research frameworks – and to provide greater options for YP in accessing help[14].

MoodHwb is a bilingual (English and Welsh), interactive, and multi-platform digital programme designed to support YP with their mood and wellbeing - and their families/carers, friends, and

practitioners (**figure 1**).

FIGURE 1 HERE

It was co-designed using a series of interviews and focus groups with YP experiencing or at high risk of depressive symptoms (due to family history), parents/carers, and professionals, alongside workshops with a digital media company and an animator[15-17]. The development was also informed by a systematic review[18], design, educational and psychological theories, and a preliminary 'logic model' (programme theory)(**figure 2**).

FIGURE 2 HERE

As part of the developmental work, there was an early evaluation of MoodHwb with a small number of YP and their parents/carers and practitioners, recruited via primary and secondary Child and Adolescent Mental Health Services (CAMHS), school counsellors and nurses, and a previous study sample (Early Prediction of Adolescent Depression (EPAD) study)[19]. Preliminary results were encouraging, and participants found the programme acceptable to use and gave feedback on how it could be improved. Findings also provided initial support for the programme theory, particularly regarding depression literacy, self-efficacy, and depressive symptoms. In line with guidance for complex interventions, a larger feasibility trial is now required[20,21].

The primary aims of the current study are to further refine and evaluate the feasibility, acceptability, and potential impact of the digital programme MoodHwb and the feasibility and acceptability of the trial methods.

METHODS AND ANALYSIS

Initial development phase

The first phase involves the further refinement of the digital programme, based on the feedback provided by participants during the earlier evaluation. The key revisions recommended included improving the navigation, audiovisual and interactive elements, making it more personalised based on user preferences, developing the app version, and expanding the self-help and anxiety

For peer review only

content[19]. There were subsequent consultations with practitioners at the proposed trial recruitment centres across Wales and Scotland, and researchers in the field.

Regular updates are required to the design and content of digital programmes because of the fast-moving pace of the field and the emerging evidence and guidelines. We have completed reviews of the co-design of digital technologies with children and YP[17] and technologies to support YP with depression and anxiety[14]. The principal investigator (PI) has visited research centres in youth and digital mental health in New Zealand, Australia, and the Republic of Georgia. Workshops and meetings will be held with the digital media company and animator that helped to develop the original prototype.

As part of a pre-trial acceptability phase, YP in Wales and Scotland (n=10-15) will be given access to the new prototype. They will be recruited through school counsellors, charities, youth advisory groups (YAGs) and self-referrals. Adolescents will be eligible to take part if they are aged 13-19 years and have sufficient understanding of the English language, access to the internet and a valid email address. They will be interviewed to ensure that the changes made are acceptable ahead of the trial, and to explore issues of transferability in Scotland, given that the programme was developed in Wales. We will aim to recruit some YP who have experienced depressive symptoms. However, others with no experience of mental health difficulties will also give feedback e.g., on whether the programme is user-friendly and age-appropriate. Practitioners from health, education and youth services will be consulted further regarding the new prototype through meetings and online communication.

Trial design

The feasibility study is a multi-centre feasibility randomised controlled trial (RCT) comparing the MoodHwb programme plus usual care (if the young person is receiving support) to digital information sheets on mood and wellbeing plus usual care (if receiving support)(**figure 3**). The research plan follows guidance for complex interventions[20,21], and digital person-centred interventions[22].

FIGURE 3 HERE

For peer review only

Setting and participants

Trial participants will be identified by practitioners in schools (by e.g., counsellors, nurses, teachers), primary mental health centres, charities, youth services and sports academies in Wales, and in schools and charities via the second site at the University of Glasgow, Scotland. The School Health Research Network (SHRN, Wales) and Schools Health and Wellbeing Improvement Research Network (SHINE, Scotland) will be involved. These sources were chosen as the developmental work suggested that the most appropriate setting for the programme would be the time when depressive difficulties start and YP first present - and is therefore a form of early intervention[16,19]. We will also recruit self-referrals, for example through social media and online advertising, including through the National Centre for Mental Health (NCMH), Wolfson Centre for YP's Mental Health (WCYPMH) and TRIUMPH youth mental health network.

Young people will be eligible if they: (1) are aged 13-19 years, (2) are experiencing depressive symptoms, (3) have a sufficient understanding of English to complete outcomes measures, (4) have access to the internet and a valid email address, and (5) live in Wales or Scotland. Exclusion criteria are: (1) already receiving specialist treatment for depression (e.g., cognitive behavioural therapy (CBT) in secondary CAMHS), and (2) the presence of other mental health difficulties where alternative support might be appropriate. Parents and carers will be targeted for the trial if they have an adolescent with depressive symptoms. Professionals will be approached if they work with YP who present with mental health difficulties.

For peer review only

Sample size

We aim to recruit up to 120 adolescent participants, and their parents/carers. Approximately two-thirds of the participants will be from Wales and one-third from Scotland. We expect a dropout of around 30%, based on previous digital health studies and the development and early evaluation work. With a sample size of 120 we can estimate the overall expected retention rate of 0.7 to within +/-0.082 (70% +/-8.2%) and within group retention as 0.7+/-0.116 based on the width of a two-sided 95% confidence interval (CI).

Recruitment

School, mental health, youth, or charity practitioners will identify potentially eligible YP and ask them whether they are interested in participating. The practitioners will provide a study postcard which directs the YP to the bilingual study website (www.ncmh.info/digital-support-study/), where there will be details of the study and information sheets. We will ask practitioners to keep a screening log of all YP approached. There will be a link to the website via social media and online advertisements for self-referrals.

If they are interested in taking part, YP will be asked to complete an online eligibility form on the website. This will include questions on their contact details and to assess whether they meet the eligibility criteria, including items from the Mood and Feelings Questionnaire (MFQ)[23]. They would be eligible if they score 20 or over on the MFQ. If they score 29 or over, they would also be advised to seek help and we will contact their general practitioner (GP), as this indicates a 'major depressive disorder'[24]. They will not be eligible if they access specialist treatment (e.g. CBT; see exclusion criteria). These cut-offs have been used in previous trials of digital interventions for depression in YP[25,26]. The research team will then contact those who are interested to discuss the project (including consent forms and questionnaires), confirm their eligibility, respond to their questions, and ask whether their parents/carers are interested in participating.

Consent

Online consent forms are provided for the participants to complete, before completion of baseline questionnaires. If under 16 years old, YP will be asked to provide assent while their parent or legal guardian will be asked for consent. Dual consent and assent will be required for participation. Those aged 16 years or older will be able to provide their own consent. Parents/guardians and professionals who participate will also be asked to complete consent forms.

Randomisation

Participants will be randomised at a 2:1 ratio to either the digital programme, MoodHwb plus usual care, or digital information sheets on mood and wellbeing in YP plus usual care (**figure 3**). We decided to randomise at a 2:1 ratio to allow for more information to be gained about those receiving

For peer review only

the intervention. The permuted block randomisation will be computerised and stratified according to site (Wales and Scotland). This will be built into the study database overseen by the Centre for Trials Research (CTR, Cardiff University), providing remote access and preserving allocation concealment. Because of the nature of the intervention, participants will not be blind to their allocation, however data analysis will be carried out blind to allocation.

Intervention

MoodHwb is a digital programme to support YP with their mood and wellbeing, particularly those with elevated depressive symptoms. It can also be used by their families/carers, friends, and practitioners. The programme and development process are described in detail in previous publications[15-17].

It is designed to engage YP by using developmentally appropriate language, illustrations, animations, and interactive components. It aims to promote self-help, help-seeking where appropriate and social support. Whilst it was founded primarily on psychoeducation, it also includes elements of CBT, behavioural activation, positive psychology, and interpersonal, family systems and behaviour change theories. The first version was developed primarily as a website, although the interactive sections (e.g., mood diary) are also available in an 'app'. It could be used independently as a 'standalone' programme by YP (or parents/carers, friends, or professionals) or supported by another person.

From the welcome screen, the user is asked questions (e.g., regarding their mood and anxiety levels), and the answers (i) are stored in their 'profile' section, and (ii) help to signpost to the relevant subsections on the subsequent 'dashboard' screen, therefore personalising the content. Along with this 'mood diary', there are other interactive elements: a goal-setting element and a section to save links to helpful resources. Five sections were developed for YP (**figure 1**), and these cover: (i) low mood and depression, (ii) possible reasons, (iii) self-help approaches, (iv) sources of help and (v) associated health difficulties. There is a sixth section to support those who might be concerned about YP, such as parents/carers, friends, and practitioners. It is recommended that users navigate sections in this order, but they can use it in any way that is helpful for them. Each of these sections includes information, animations, personal stories, exercises, and links to relevant resources. MoodHwb is bilingual - 'hwb' is the Welsh translation for 'hub', and can mean a 'lift' or 'boost'. HwbHwyliau is the Welsh name for the programme.
Participants in the control arm will be emailed a digital information pack, which will consist of digital information sheets on mood and wellbeing (including depression) from the NCMH, Royal College of

Psychiatrists and YoungMinds websites.

Patient and public involvement

The programme was co-designed using a series of interviews (n=12) and focus groups (n=6) with (i) YP who either had a history of depression or were at elevated risk, (ii) parents/carers, and (iii) professionals. Participants were recruited from CAMHS in Wales, and families from the EPAD study. As noted, further feedback on the programme was provided during the early evaluation[19].

The project has been adopted by the Centre for Development, Evaluation, Complexity and Implementation in Public Health Improvement (DECIPHer, Cardiff University), and the study plans were developed in collaboration with their YAG, ALPHA (Advice Leading to Public Health Advancement). We will collaborate further with them as well as the YAGs of the WCYPMH, NCMH and TRIUMPH network. A young person has been invited to join the Trial Steering Committee (TSC), and other YP (aged 13 to 16 years) have reviewed study documents e.g., information sheets and consent forms. Practitioners and researchers have been consulted regarding the plans.

Assessment schedule

Data will be collected at (i) baseline and (ii) follow-up, two months after randomisation. Data will be collected from (i) baseline and follow-up online questionnaires; (ii) semi-structured interviews with YP and parents/carers who used the programme; (iii) a focus group with professionals and (iv) Web/app usage data (**table 1**).

For peer review only

Table 1: Schedule of enrolment, interventions and assessments for the trial[1]

Time point	Screening (contact 1)	Baseline (contact 1)		Month 2 (contact 2)	
**Activity**		**Inter-vention**	**Control**	**Inter-vention**	**Control**
**Referral to the trial/Register of interest**	X				
**Eligibility assessment**	X				
**Consent process**	X				
**Randomisation**	X				
**Access to MoodHwb**		X			
**Questionnaires**					
Adolescent Depression Knowledge Questionnaire (ADKQ)[27]		•/○	•/○	•/○	•/○
Depression Stigma Scale (DSS)[28]		•/○	•/○	•/○	•/○
General Self-Efficacy Scale (GSE)[29]		•	•	•	•
General Health-Seeking Questionnaire (GHSQ)[30]		•	•	•	•
Warwick-Edinburgh Mental Wellbeing Scales (WEMWBS)[31]		•/○	•/○	•/○	•/○
Mood and Feelings Questionnaire (MFQ)[23]		•/○	•/○	•/○	•/○
Revised Children's Anxiety and Depression Scale-Short Version (RCADS-25)[32]		•/○	•/○	•/○	•/○
Hospital Anxiety and Depression Scale (HADS)[33]		○	○	○	○
Items on Health economics & Life Events[34]		•/○	•/○	•/○	•/○
Items on use and acceptability of MoodHwb				•/○	
**Process evaluation**					
Young person interview (n=20-25)				•	
Parent/carer Interview (n=10-15)				○	
Professionals focus group				*	

(• participant/○ parent/*professionals) 'Contact' will comprise phone/videoconferencing discussion and completion of online forms/questionnaires. Taken from the HRA CTIMP protocol template (2016).

For peer review only - http://bmjopen.bmj.com/site/about/guidelines.xhtml

We will use remote methods for recruitment, consent, and data collection (e.g., phone, email, videoconferencing, online forms and questionnaires) because these have been shown to be efficient and effective ways of conducting research during the Covid-19 pandemic. This approach is consistent with the digital nature of the programme and would enable us to reach more YP. All participants will be offered £10 gift vouchers after (i) completing baseline questionnaires, (ii) completing follow-up questionnaires, and (iii) participating in interviews.

Baseline

At baseline, YP in both arms will complete questionnaires which will include items on their demographic and contact information, health and use of services, and standardised questionnaires on knowledge, stigma, self-efficacy, help-seeking, wellbeing, symptoms, and life stresses (**table 1**). The parent/carer questionnaires will include items on the young person's details, use of services and symptoms, and their knowledge, beliefs, wellbeing, and symptoms. All participants will also complete items on the acceptability of the questionnaires.

Follow-up

All baseline measures will be repeated at the follow-up timepoint, two months post-randomisation. In addition, those in the intervention arm will be asked about their use of the programme, and their views on the content and design. Those in the control arm will be asked whether they had access to MoodHwb, to explore contamination. A subgroup of YP (n=up to 20-25) and parents/carers (n=up to 10-15) who used the programme will be interviewed at follow-up on their use of and views of MoodHwb and the evaluation process. The Web/app usage of those using the programme will be monitored using Google Analytics up to 6 months post-randomisation, to provide information on longer-term use. There will be a focus group for professionals who work with YP from a range of settings, to ascertain their views.

For peer review only

Process evaluation

The interviews with YP and parents/carers at follow-up and practitioner focus group noted earlier will provide more in-depth information on the acceptability and feasibility of the programme and evaluation process – in addition to the questionnaire and Web/app usage data. Participants will be purposively selected for interview based on their usage of MoodHwb (i.e., frequent or occasional), age, gender, support received (e.g. school, health service), and primary language (English, Welsh) – so that there is a broad range of viewpoints. A process evaluation framework has been developed, following MRC guidance[35], and focuses primarily on the intervention but also examines certain trial processes. It addresses issues regarding reach, contamination, retention, recruitment, context, exposure, and fidelity.

Outcome measures

Primary outcome

The feasibility and acceptability of MoodHwb outcomes will include the level of usage and adherence (including number of times accessed and duration of use), views and acceptability of the design and content (including strengths, areas to develop further and approaches to integrate into services and charities), and technical and accessibility aspects. The feasibility and acceptability outcomes of the trial methods will include recruitment and retention rates, completeness of measures, representativeness, randomisation, contamination, and acceptability (particularly of the remote methods). Progression criteria for a full trial have been developed with the TSC, based on these outcomes (**appendix 1**).

Secondary outcomes

Secondary outcomes assess the potential impact of the programme on a core set of domains, including depression literacy or knowledge, depression stigma, help-seeking behaviour, self-efficacy, wellbeing, and depression and anxiety symptoms. These will be measured via standardised validated questionnaires (**table 1**), and participant feedback (via questionnaires and interviews).

For peer review only

Exploratory economic evaluation

We will investigate the feasibility and acceptability of collecting health economic data. We will identify and measure the resources involved in intervention delivery as well as resource use by participants, with reference to methodology from health economics guidance[36,37]. An estimation of the following will be collected: i) intervention costs (development, hosting, and maintenance costs), ii) health (e.g., contact with GPs, hospitals and other health settings, medication), social, educational, and personal resource costs, using questionnaire items adapted from the Client Service Receipt Inventory, iii) wellbeing, using WEMWBS[31].

For peer review only

Planned analysis

Primary outcome findings will be derived primarily from analysis of the questionnaires, interviews, and focus group, with additional analysis of Web/app usage data regarding usage, adherence and technical aspects, and screening logs and participant database regarding recruitment and retention. Secondary outcome findings will be derived primarily from analysis of validated questionnaires.

Participant and Web usage data

The flow of participants through the trial will be presented as a CONSORT flowchart[38]. Baseline demographic data will be presented for all consented and randomised participants. This will be summarised and tabulated for the whole group and by arm, using mean (SD) or median (IQR) where appropriate. All subsequent summaries will use complete cases and will employ the intention to treat principle (participants will remain in groups to which they were randomised irrespective of intervention received). Web usage data from Google Analytics will be tabulated, for example related to number of times accessed, duration of use, sections accessed, and language.

Qualitative analysis

The interviews and focus group recordings will be transcribed, and data analysis will proceed using a thematic analysis approach. This is a process of identifying, analysing, reporting, and interpreting

patterns or themes, and is one of the most used qualitative approaches in health research[39]. Codes will be applied to broad themes and then broken down further into sub-codes - 30% will be double-coded to ensure the reliability of coding, and differences will be resolved through discussion. Transcripts will be closely examined to identify key themes and sub-themes. Thematic analysis will be supported by the computer-assisted qualitative analysis software, NVivo.

Quantitative analysis

Baseline questionnaire data will be scored according to the validated scoring algorithms from the relevant literature. Missing item-by-item data will be handled according to the scoring algorithm for each measure individually. Baseline scores will be presented descriptively, using summary statistics (means, SD, medians, IQR and percentages with associated 95% CI where appropriate) for the whole group and by arm. Distributional assumptions will be checked, and data transformation carried out if required. A comparison of randomised groups will be tabulated but not tested, in line with the feasibility aims. A comparison of baseline data for those completing and not completing the study will be tabulated to assess drop-out bias.

Scores at the two-month time point will be tabulated and compared with baseline in a complete case analysis. No hypothesis tests will be carried out but 95% CI for the difference at two months between the two arms will be estimated and reported. These will be adjusted for baseline score and any factors such as site used to balance the randomisation. Statistical analysis will be performed using Stata software, but no p-values will be presented. We will further develop and test the MoodHwb logic model (**figure 2**) using multiple methods, and examine possible mechanisms of action and active components. This will help to define a core set of outcome measures (related to the model) and identify the most appropriate primary outcome for evaluation in an effectiveness trial.

Trial management

A Trial Management Group (TMG) and an independent TSC have been established to monitor progress and advise the researchers. The TMG will meet approximately every 8-10 weeks during the trial planning and throughout the trial. The TMG is composed of the PI, co-investigators, research assistant, trial administrator, CTR members, and University of Glasgow site team. The TSC will meet

For peer review only

approximately every 12 months. The TSC includes a young person, and an independent digital mental health researcher, statistician, and mixed-methods researcher.

Adverse event reporting and harms

For this trial, adverse events (AEs) are defined as any untoward medical occurrence in a participant administered an intervention and which are not necessarily caused by or related to that intervention. This would include significant deterioration in mental health symptoms, including depressive and anxiety symptoms, and emergence of thoughts of self-harm. Serious adverse events (SAEs) include those resulting in death, are life-threatening, require hospitalisation or prolongation of existing hospitalisation, result in persistent or significant disability or incapacity, or other medically important conditions. The PI will assess each SAE for expectedness. A standard study/departmental procedure for documenting and dealing appropriately with these events will be followed. Participants will be signposted to appropriate resources and services if required, and their GP details will be collected at baseline, so they can be contacted. An urgent safety measure is an action that the Sponsor or PI may carry out to protect the participants of a trial against any immediate hazard to their health or safety – and must be notified to the REC immediately.

Ethics and dissemination

Approvals were received from the Cardiff University School of Medicine REC and University of Glasgow College of Medical, Veterinary and Life Sciences REC for the pre-trial acceptability phase with YP. Favourable ethical opinion for the overall study and trial was obtained from Wales REC 3 (reference 21/WA/0205), and approvals were received by HRA, HCRW, university health board R&D departments in Wales, and schools in Wales (including via SHRN) and Scotland (including via SHINE). The study was registered with ISRCTN (ISRCTN12437531). Approvals were received for MoodHwb to be added to the (Apple) App Store and Google Play App Store.

We will disseminate our findings to academics, researchers, policymakers, and practitioners through high-impact open-access publications and presentations at relevant academic, clinical, and educational conferences and meetings. The findings will be disseminated online, for example on the NCMH, WCYPMH and TRIUMPH websites. Results will be made available to all participants after the study completion.

For peer review only

DISCUSSION

MoodHwb is a digital programme to support YP with their mood and wellbeing, especially those experiencing elevated depressive symptoms, and families/carers, friends, and professionals who are involved in supporting them. It can be used as a website and as an app, as a standalone programme or supported by others, for example in counselling or therapy sessions. Digital mental health technologies such as this have the potential to improve reach and access to therapies at a relatively low cost. MoodHwb will help to address the need for accessible, evidence-based technologies that are developed with user input and evaluated rigorously – and will provide YP and others with more options when looking for support.

Strengths and limitations

MoodHwb was co-designed with YP with lived experience or at high risk of depression, parents/carers, and practitioners. The programme is bilingual, interactive, personalised according to user needs and preferences, and multi-platform. It has been developed in line with evidence-based approaches, not only for YP but also for those concerned about them. Multiple methods will be used to collect data in a range of settings, including validated questionnaires, semi-structured interviews, a focus group, and Web/app usage monitoring. The involvement of participants in the second site in Scotland will help to boost recruitment and enable the exploration of transferability.

Given that this is a feasibility study, we will not be able to explore effectiveness; however the information and experience will inform the design of a future effectiveness trial. Whilst it is not feasible to blind participants to allocation; data analysis will be carried out blind to allocation. We have minimised the risk of contamination between trial arms by ensuring that a log-in is required to access MoodHwb. There will be items in the follow-up questionnaire for participants in the control arm on whether they have accessed MoodHwb during the study.

At the end of the study, there will be a period of reflection and planning regarding what further development, evaluation and implementation work are required for MoodHwb, both as a standalone and supported technology, in collaboration with YP. If it is ultimately found to be feasible and effective, it could be rolled out to help YP, families/carers, and practitioners across a range of settings.

For peer review only

Trial status

Recruitment started in September 2022 and will end in May 2023.

Trial sponsor

Cardiff University is the Sponsor of the study.

Authors' contributions

RBJ designed and obtained funding for the study, with research support from SAS, FR, SM, and PS and advice from ER, RM, RP, SC, VP, and RWT (CTR). RBJ drafted the initial study protocol. All authors contributed to and approved the final version.

Funding statement

This work was supported by a National Institute for Health Research (NIHR) and Health and Care Research Wales (HCRW) Post Doctoral Fellowship programme, grant number NIHR-PDF-2018, awarded to RBJ. SS was supported by UK MRC and Scottish Chief Scientist Office core funding as part of the MRC/CSO Social and Public Health Sciences Unit 'Complexity in Health Improvement' programme (MC_UU_12017/14 and SPHSU14) and MC-PC_13027. The work was also supported by the NCMH, WCYPMH (established with support from the Wolfson Foundation) and TRIUMPH network.

Acknowledgements

The authors would like to thank all the YP, parents, carers, professionals, and researchers who participated in the development of this protocol. We would like to thank Caroline Warren, Gareth Watson, and Tim Pickles (all Cardiff University) for their contribution to the trial administration, database and statistics respectively, and Emma McIntosh and Kathleen Boyd (both University of Glasgow) for their contributions to the health economics. We thank the TSC for their support.

For peer review only

Competing interests statement

The authors declare they have no competing or potential conflicts of interest.

REFERENCES

1. Thapar, A., Eyre, O., Patel, V., Brent, D., 2022. Depression in young people. Lancet 400, 617–631. https://doi.org/https://doi.org/10.1016/S0140-6736(22)01012-1

2. Knapp, M., & Wong, G. (2020). Economics and mental health: the current scenario. *World Psychiatry : Official Journal of the World Psychiatric Association (WPA)*, *19*(1), 3–14. https://doi.org/10.1002/WPS.20692

3. Trautmann, S., Rehm, J., & Wittchen, H. (2016). The economic costs of mental disorders: Do our societies react appropriately to the burden of mental disorders? *EMBO Reports*, *17*(9), 1245. https://doi.org/10.15252/EMBR.201642951

4. Potter, R., Mars, B., Eyre, O., Legge, S., Ford, T., Sellers, R., … Thapar, A. K. (2012). Missed opportunities: mental disorder in children of parents with depression. *The British Journal of General Practice : The Journal of the Royal College of General Practitioners*, *62*(600), e487-93. https://doi.org/10.3399/bjgp12X652355

5. Neufeld, S. A. S., Dunn, V. J., Jones, P. B., Croudace, T. J., & Goodyer, I. M. (2017). Reduction in adolescent depression after contact with mental health services: a longitudinal cohort study in the UK. *The Lancet. Psychiatry*, *4*(2), 120–127. https://doi.org/10.1016/S2215-0366(17)30002-0

6. Bennett, C., Jones, R. B., & Smith, D. (2014). Prevention strategies for adolescent depression. *Advances in Psychiatric Treatment*, *20*(2), 116–124. https://doi.org/10.1192/apt.bp.112.010314

7. National Institute for Health and Clinical Excellence (NICE). (2019). Depression In Children and Young People. *NICE Clinical Guideline*. Retrieved from http://guidance.nice.org.uk/CG28/Guidance

8. Birmaher, B., Brent, D., 2007. Practice Parameter for the Assessment and Treatment of Children and Adolescents With Depressive Disorders. J. Am. Acad. Child Adolesc. Psychiatry 46, 1503–1526. https://doi.org/10.1097/CHI.0B013E318145AE1C

9. Office for National Statistics. (2019). Exploring the UK's digital divide - Office for National Statistics. *Office for National Statistics*, 1–24.

For peer review only

10. Ofcom. (2021). Online Nation 2021, (June), 16. Retrieved from https://www.ofcom.org.uk/__data/assets/pdf_file/0013/220414/online-nation-2021-report.pdf

11. Naslund, J. A., Aschbrenner, K. A., Araya, R., Marsch, L. A., Unützer, J., Patel, V., & Bartels, S. J. (2017). Digital technology for treating and preventing mental disorders in low-income and middle-income countries: a narrative review of the literature. *The Lancet Psychiatry*, *4*(6), 486–500. https://doi.org/10.1016/S2215-0366(17)30096-2

12. Hollis, C., Falconer, C. J., Martin, J. L., Whittington, C., Stockton, S., Glazebrook, C., & Davies, E. B. (2017). Annual Research Review: Digital health interventions for children and young people with mental health problems-a systematic and meta-review. 58(4): 474-503. https://doi.org/10.1111/jcpp.12663

13. Grist, R., Croker, A., Denne, M., & Stallard, P. (2019). Technology Delivered Interventions for Depression and Anxiety in Children and Adolescents: A Systematic Review and Meta-analysis. *Clinical Child and Family Psychology Review*, *22*(2), 147–171. https://doi.org/https://dx.doi.org/10.1007/s10567-018-0271-8

14. Bevan Jones, R., Hussain, F., Agha, S. S., Weavers, B., Lucassen, M., Merry, S., … Rice, F. (2022). Digital technologies to support adolescents with depression and anxiety: review. *BJPsych Advances*, 1–15. https://doi.org/DOI: 10.1192/bja.2022.3

15. Bevan Jones, R., Thomas, J., Lewis, J., Read, S., All, I. J.-R. for, & 2017, U. (2017). Translation: from bench to brain–using the visual arts and metaphors to engage and educate. *Scienceopen.Com*, *1*(2), 265–283. https://doi.org/10.18546/RFA.01.2.04

16. Bevan Jones, R., Thapar, A., Rice, F., Beeching, H., Cichosz, R., Mars, B., … Simpson, S. A. (2018b). A web-based psychoeducational intervention for adolescent depression: design and development of MoodHwb. *JMIR Mental Health*, *5*(2). https://doi.org/10.2196/mental.8894

17. Bevan Jones, R., Stallard, P., Agha, S. S., Rice, S., Werner-Seidler, A., Stasiak, K., … Merry, S. (2020b). Practitioner review: Co-design of digital mental health technologies with children and young people. *Journal of Child Psychology and Psychiatry*, *61*(8), 928–940. https://doi.org/10.1111/JCPP.13258

18. Bevan Jones, R., Thapar, A., Stone, Z., Thapar, A., Jones, I., Smith, D., & Simpson, S. (2018a). Psychoeducational interventions in adolescent depression: A systematic review. *Patient Education and Counseling*, *101*(5), 804–816. https://doi.org/10.1016/J.PEC.2017.10.015

19. Bevan Jones, R., Thapar, A., Rice, F., Mars, B., Agha, S. S., Smith, D., … Simpson, S. A. (2020a). A Digital Intervention for Adolescent Depression (MoodHwb): Mixed Methods Feasibility Evaluation. *JMIR Ment Health 2020;7(7):E14536 Https://Mental.Jmir.Org/2020/7/E14536*, *7*(7), e14536. https://doi.org/10.2196/14536

For peer review only

20. Craig, P., Dieppe, P., Macintyre, S., Mitchie, S., Nazareth, I., & Petticrew, M. (2008). Developing and evaluating complex interventions: the new Medical Research Council guidance. *BMJ*, *337*(7676), 979–983. https://doi.org/10.1136/BMJ.A1655

21. Skivington, K., Matthews, L., Simpson, S.A., Craig, P., Baird, J., Blazeby, J.M., Boyd, K.A., Craig, N., French, D.P., McIntosh, E., Petticrew, M., Rycroft-Malone, J., White, M., Moore, L., 2021. A new framework for developing and evaluating complex interventions: update of Medical Research Council guidance. BMJ 374. https://doi.org/10.1136/bmj.n2061

22. Yardley, L., Morrison, L., Bradbury, K., & Muller, I. (2015). The Person-Based Approach to Intervention Development: Application to Digital Health-Related Behavior Change Interventions. *J Med Internet Res 2015;17(1):E30 Https://Www.Jmir.Org/2015/1/E30*, *17*(1), e4055. https://doi.org/10.2196/JMIR.4055

23. Angold, A., Costello, E., Messer SC, & Pickles A. (1995). Development of a short questionnaire for use in epidemiological studies of depression in children and adolescents. *International Journal of Methods in Psychiatric Research*. Retrieved from https://psycnet.apa.org/record/1996-02633-002

24. Daviss, W., Birmaher, B., Melhem, N. A., Axelson, D. A., Michaels, S. M., & Brent, D. A. (2006). Criterion validity of the Mood and Feelings Questionnaire for depressive episodes in clinic and non-clinic subjects. *Journal of Child Psychology and Psychiatry and Allied Disciplines*, *47*(9), 927–934. https://doi.org/10.1111/j.1469-7610.2006.01646.x

25. Smith, P., Scott, R., Eshkevari, E., Jatta, F., Leigh, E., Harris, V., … Yule, W. (2015). Computerised CBT for depressed adolescents: Randomised controlled trial. *Behaviour Research and Therapy*, *73*, 104–110. https://doi.org/10.1016/J.BRAT.2015.07.009

26. Wright, B., Tindall, L., Hargate, R., Allgar, V., Trépel, D., & Ali, S. (2019). Computerised cognitive-behavioural therapy for depression in adolescents: 12-month outcomes of a UK randomised controlled trial pilot study. *BJPsych Open*, *6*(1). https://doi.org/10.1192/BJO.2019.91

27. Hart, S. R., Kastelic, E. A., Wilcox, H. C., Mary, •, Beaudry, B., Musci, R. J., … Swartz, K. L. (2014). Achieving Depression Literacy: The Adolescent Depression Knowledge Questionnaire (ADKQ). https://doi.org/10.1007/s12310-014-9120-1

28. Griffiths, K. M., Christensen, H., Jorm, A. F., Evans, K., & Groves, C. (2004). Effect of web-based depression literacy and cognitive-behavioural therapy interventions on stigmatising attitudes to depression: randomised controlled trial. *The British Journal of Psychiatry : The Journal of Mental Science*, *185*(OCT.), 342–349. https://doi.org/10.1192/BJP.185.4.342

29. Schwarzer, R., & Jerusalem, M. (1995). General Self-Efficacy Scale. *J. Weinman, S. Wright, & M. Johnston, Measures in Health Psychology: A User's Profile. Causal and Control Beliefs*, *35*. Retrieved from https://psycnet.apa.org/doiLanding?doi=10.1037%2Ft00393-000

For peer review only

30. Wilson, C. J., Deane, F. P., Ciarrochi, J., & Rickwood, D. (2005). Measuring Help-Seeking Intentions: Properties of the General Help-Seeking Questionnaire. *Canadian Journal of Counselling*, *39*(1), 15–28. Retrieved from https://psycnet.apa.org/record/2005-05989-002

31. Tennant, R., Hiller, L., Fishwick, R., Platt, S., Joseph, S., Weich, S., … Stewart-Brown, S. (2007). The Warwick-Edinburgh mental well-being scale (WEMWBS): Development and UK validation. *Health and Quality of Life Outcomes*, *5*(1), 1–13. https://doi.org/10.1186/1477-7525-5-63/TABLES/4

32. Ebesutani, C., Korathu-Larson, P., Nakamura, B. J., Higa-McMillan, C., & Chorpita, B. (2017). The Revised Child Anxiety and Depression Scale 25–Parent Version: Scale Development and Validation in a School-Based and Clinical Sample. *Assessment*, *24*(6), 712–728. https://doi.org/10.1177/1073191115627012

33. Zigmond, A. S., & Snaith, R. P. (1983). The Hospital Anxiety and Depression Scale. *Acta Psychiatrica Scandinavica*, *67*(6), 361–370. https://doi.org/10.1111/J.1600-0447.1983.TB09716.X

34. Johnson, J. H., & McCutcheon, S. M. (1980). Assessing life stress in older children and adolescents: Preliminary findings with the Life Events Checklist. *Stress and anxiety*, *7*, 111-125.

35. Moore, G. F., Audrey, S., Barker, M., Bond, L., Bonell, C., Hardeman, W., … Baird, J. (2015). Process evaluation of complex interventions: Medical Research Council guidance. *BMJ (Clinical Research Ed.)*, *350*, h1258. https://doi.org/10.1136/bmj.h1258

36. National Institute for Health and Clinical Excellence (NICE). (2012). 6 Incorporating health economics | Methods for the development of NICE public health guidance (third edition) | Guidance | NICE. Retrieved March 1, 2022, from https://www.nice.org.uk/process/pmg4/chapter/incorporating-health-economics

37. Tudor Edwards, R., & McIntosh, E. (2019). *Applied Health Economics for Public Health Practice and Research* (1st ed.). Oxford, UK: Oxford University Press. https://doi.org/10.1093/med/9780198737483.001.0001

38. Rennie, D. (2001). CONSORT revised--improving the reporting of randomized trials. JAMA 285, 2006–2007. https://doi.org/10.1001/JAMA.285.15.2006

39. Pope, C., & Mays, N. (2006). *Qualitative methods in health research* (Vol. 5). Blackwell Publishing: BMJ Books.

For peer review only

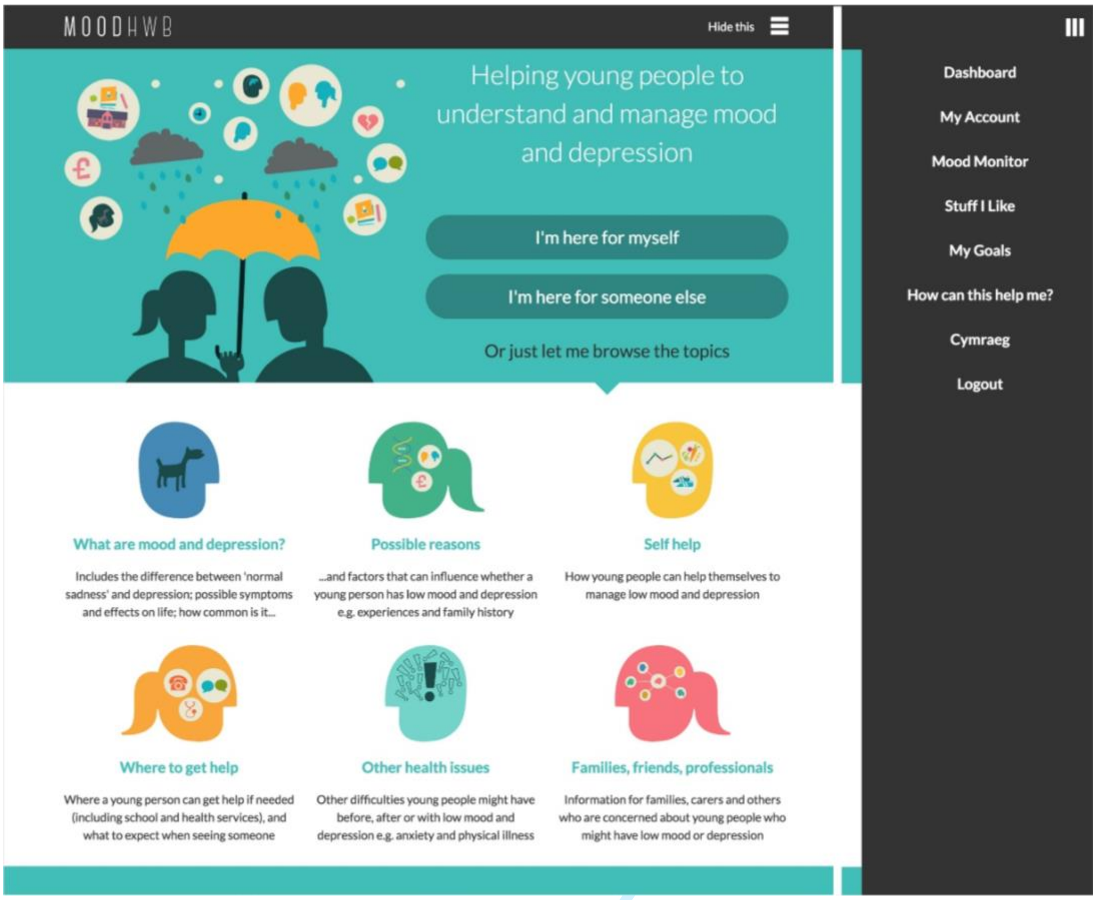

Figure 1:

MoodHwb (v1) welcome screen (main image/left) and open menu (right)

(from Bevan Jones et al. 2020a)

For peer review only - http://bmjopen.bmj.com/site/about/guidelines.xhtml

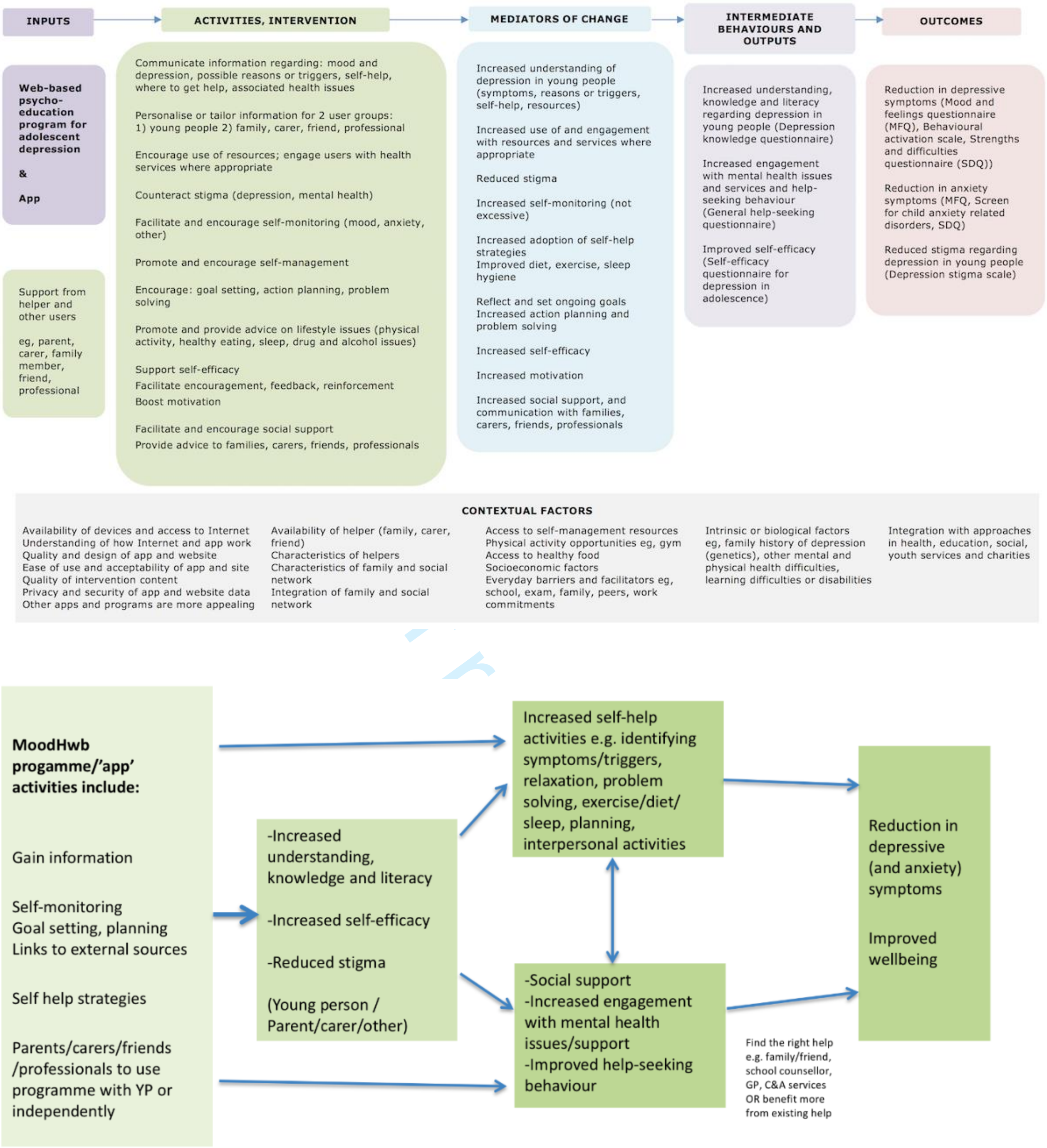

Figure 2:

Logic model for MoodHwb (above, from Bevan Jones et al 2018b), including potential pathways (below)

(applies primarily to the young person using the programme, although other users (parent/carer/other) might also benefit from the programme through e.g. increased depression literacy/knowledge or reduced stigma)

Figure 3: Participant flow diagram

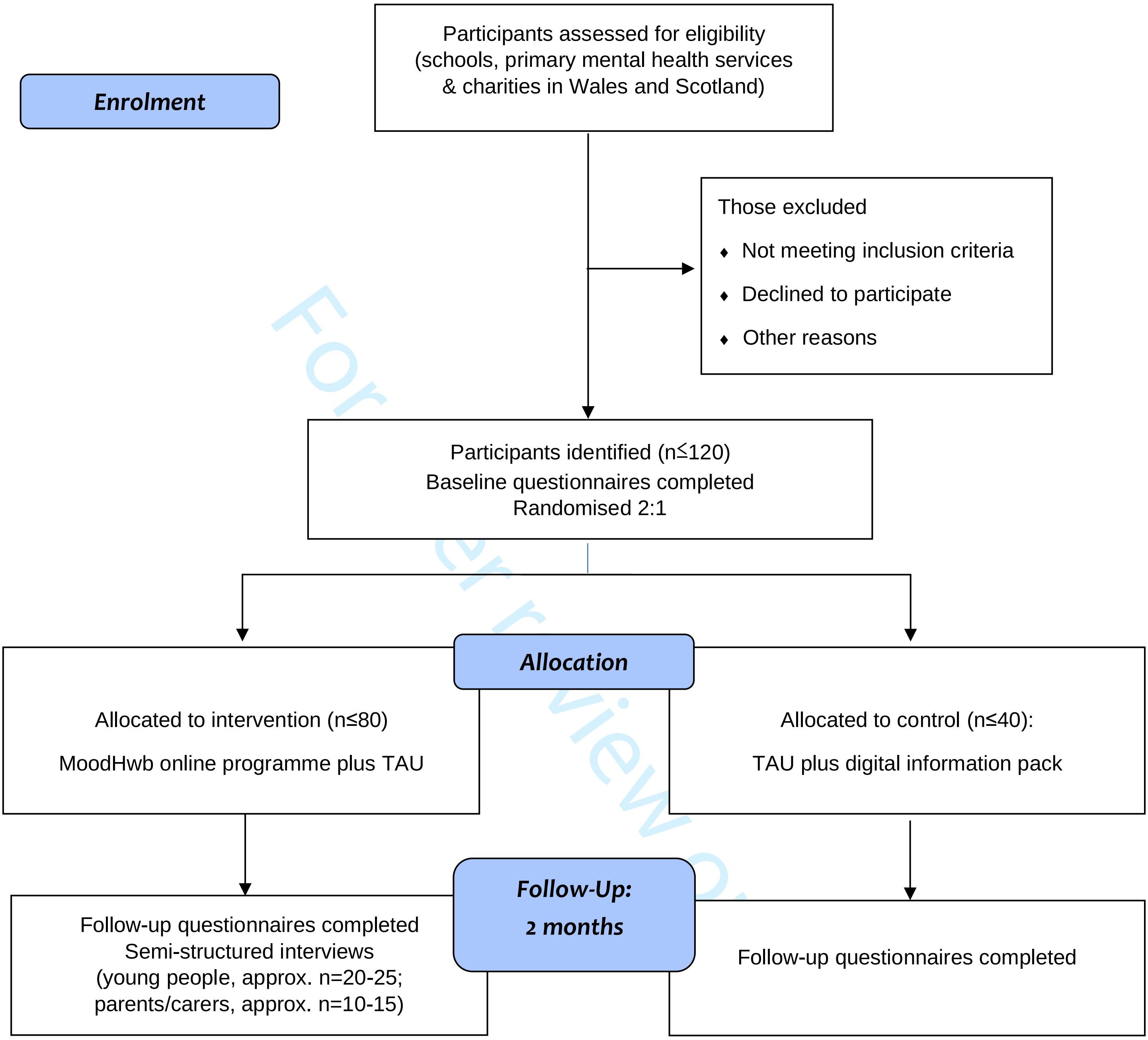

NB: Web/app usage data of those in the intervention arm will collected for 6 months after baseline.

For peer review only - http://bmjopen.bmj.com/site/about/guidelines.xhtml

Proposed progression criteria – 'Digital support for young people with their mood and wellbeing' study

**CRITERIA**	**INDICATOR**<br>GREEN = very strong indication to proceed<br>AMBER = medium indication to proceed. Discuss with Trial Steering Committee (TSC) and proceed with identified plan.<br>RED = indication of doubt as to whether to proceed. Discuss with TSC, and only proceed if other indicators are amber/green and there is a clear mitigation strategy.	**METHOD OF ASSESSMENT**
**1. Feasibility and acceptability outcomes related to the trial methods**		
1.1. Recruitment rate	Actual recruitment rate vs. Target recruitment rate:<br>Green: ≥85% of participants<br>Amber: 60-84%<br>Red: <60%	Number of eligible young people who consent to participate in the study within the first 6 months of recruitment<br>Vs. Target number (120) within this period<br><br>[Also to review % of young people who showed an interest in the study who go on to participate]
1.2. Retention rate	Retention rates:<br>Green: ≥75% of participants<br>Amber: 50-74%<br>Red: <50%	Number of young people who remained in the study at 2 months<br>Vs. Total number who consented to participate at baseline
1.3. Completeness of outcome measures	Completion of core measures at baseline and follow-up:<br>Green: ≥90% data completion	% of participants who completed the core questionnaires (including measures on

For peer review only - http://bmjopen.bmj.com/site/about/guidelines.xhtml

	Amber: 70-89%<br>Red: <70%	depressive and anxiety symptoms, wellbeing, knowledge and help-seeking)<br>% of missing data from completed core questionnaires<br><br>Both at i) baseline and ii) 2-month follow-up (i.e. based on completion by those who remained in the study)<br><br>[Also to review views/acceptability of trial methods from questionnaire, interview and focus group data]
**2. Feasibility and acceptability outcomes related to the digital programme**		
2.1. Level of usage of programme	% of participants in the intervention arm who accessed and used the programme:<br>Green: ≥80%<br>Amber: 60-79%<br>Red: <60%<br><br>Also to discuss number of times the programme has been accessed, duration of use and the sections/components accessed.	Web/app usage data from Google Analytics, based on log-ins into and use of MoodHwb by young people (and parents/carers) – up to 2 and 6 months from baseline<br><br>Questionnaire items on usage completed by young people and parents/carers at 2-month follow-up<br><br>Semi-structured interviews with young people and parents/carers at 2-month follow-up
2.2. Views or acceptability of design and content of programme	Progression to be agreed in conjunction with TSC based on data captured around design, content, and technical/accessibility aspects of MoodHwb.	Qualitative data from semi-structured interviews with young people and parents/carers after 2 months

For peer review only

	Green: If changes are needed, they are minor or have been overcome over the course of the study<br>Amber: Changes needed are significant, but feasible with further development work<br>Red: Changes needed are significant and not feasible to address	Qualitative data from focus group with professionals<br><br>Questionnaire items on acceptability completed by young people and parents/carers at 2-month follow-up<br><br>Web/app usage data from Google Analytics (e.g. on technical/accessibility aspects), based on young person and parent/carer use of MoodHwb - up to 6 months after baseline
**3. Outcome measures for a full trial**		
3.1 Identification of primary outcomes for full trial	Discussion on the determination of primary outcome measures with TSC, including reflection on efficacy signals from candidate outcomes	Questionnaire data at 2 month follow-up<br><br>Semi-structured interviews with young people and parents/carers at 2-month follow-up<br><br>Focus group with professionals

For peer review only

STANDARD PROTOCOL ITEMS: RECOMMENDATIONS FOR INTERVENTIONAL TRIALS

SPIRIT 2013 Checklist: Recommended items to address in a clinical trial protocol and related documents*

Notes on 'Further development and feasibility randomised control trial of a digital programme for adolescent depression, MoodHwb: study protocol' (submitted to BMJ Open) in blue below. Please contact the corresponding author for further information.

Section/item	Item No	Description
**Administrative information**		
Title	1	Descriptive title identifying the study design, population, interventions, and, if applicable, trial acronym<br><br>See title page
Trial registration	2a	Trial identifier and registry name. If not yet registered, name of intended registry
	2b	All items from the World Health Organization Trial Registration Data Set<br><br>See abstract and 'Ethics and disseminations' section. Trial registration number: ISRCTN12437531
Protocol version	3	Date and version identifier<br><br>This is the first submission of the protocol for publication and is based on v6 of the trial protocol.
Funding	4	Sources and types of financial, material, and other support<br><br>See 'Funding statement'
Roles and responsibilities	5a	Names, affiliations, and roles of protocol contributors<br><br>See title page, and 'Authors' contributions'
	5b	Name and contact information for the trial sponsor<br><br>See 'Trial sponsor' section. Contact information can be requested from the corresponding author.

For peer review only

	5c	Role of study sponsor and funders, if any, in study design; collection, management, analysis, and interpretation of data; writing of the report; and the decision to submit the report for publication, including whether they will have ultimate authority over any of these activities<br><br>The sponsor and funders did not have a role in these activities
	5d	Composition, roles, and responsibilities of the coordinating centre, steering committee, endpoint adjudication committee, data management team, and other individuals or groups overseeing the trial, if applicable (see Item 21a for data monitoring committee)<br><br>See 'Trial management' section
**Introduction**		
Background and rationale	6a	Description of research question and justification for undertaking the trial, including summary of relevant studies (published and unpublished) examining benefits and harms for each intervention<br><br>See 'Background' section
	6b	Explanation for choice of comparators<br><br>See 'Trial design' and 'Intervention' sections
Objectives	7	Specific objectives or hypotheses<br><br>See 'Background' section
Trial design	8	Description of trial design including type of trial (eg, parallel group, crossover, factorial, single group), allocation ratio, and framework (eg, superiority, equivalence, noninferiority, exploratory)<br><br>See 'Trial design' section
**Methods: Participants, interventions, and outcomes**		
Study setting	9	Description of study settings (eg, community clinic, academic hospital) and list of countries where data will be collected. Reference to where list of study sites can be obtained<br><br>See 'Setting and participants' section
Eligibility criteria	10	Inclusion and exclusion criteria for participants. If applicable, eligibility criteria for study centres and individuals who will perform the interventions (eg, surgeons, psychotherapists)<br><br>See 'Setting and participants' section

For peer review only

Interventions	11a	Interventions for each group with sufficient detail to allow replication, including how and when they will be administered<br><br>See 'Intervention' section
	11b	Criteria for discontinuing or modifying allocated interventions for a given trial participant (eg, drug dose change in response to harms, participant request, or improving/worsening disease)<br><br>See 'Adverse event reporting and harms'
	11c	Strategies to improve adherence to intervention protocols, and any procedures for monitoring adherence (eg, drug tablet return, laboratory tests)<br><br>n/a
	11d	Relevant concomitant care and interventions that are permitted or prohibited during the trial<br><br>See reference to 'usual care' throughout – especially in 'Setting and participants'
Outcomes	12	Primary, secondary, and other outcomes, including the specific measurement variable (eg, systolic blood pressure), analysis metric (eg, change from baseline, final value, time to event), method of aggregation (eg, median, proportion), and time point for each outcome. Explanation of the clinical relevance of chosen efficacy and harm outcomes is strongly recommended<br><br>See 'Outcome measures' section
Participant timeline	13	Time schedule of enrolment, interventions (including any run-ins and washouts), assessments, and visits for participants. A schematic diagram is highly recommended (see Figure)<br><br>See 'Table 1' and 'Figure 3'
Sample size	14	Estimated number of participants needed to achieve study objectives and how it was determined, including clinical and statistical assumptions supporting any sample size calculations<br><br>See 'Sample size' section
Recruitment	15	Strategies for achieving adequate participant enrolment to reach target sample size<br><br>See 'Recruitment' section

Methods: Assignment of interventions (for controlled trials)

Allocation:

Sequence generation	16a	Method of generating the allocation sequence (eg, computer-generated random numbers), and list of any factors for stratification. To reduce predictability of a random sequence, details of any planned restriction (eg, blocking) should be provided in a separate document that is unavailable to those who enrol participants or assign interventions
Allocation concealment mechanism	16b	Mechanism of implementing the allocation sequence (eg, central telephone; sequentially numbered, opaque, sealed envelopes), describing any steps to conceal the sequence until interventions are assigned
Implementation	16c	Who will generate the allocation sequence, who will enrol participants, and who will assign participants to interventions
Blinding (masking)	17a	Who will be blinded after assignment to interventions (eg, trial participants, care providers, outcome assessors, data analysts), and how
	17b	If blinded, circumstances under which unblinding is permissible, and procedure for revealing a participant's allocated intervention during the trial
		All of the above – see 'Randomisation' section

Methods: Data collection, management, and analysis

Data collection methods	18a	Plans for assessment and collection of outcome, baseline, and other trial data, including any related processes to promote data quality (eg, duplicate measurements, training of assessors) and a description of study instruments (eg, questionnaires, laboratory tests) along with their reliability and validity, if known. Reference to where data collection forms can be found, if not in the protocol
	18b	Plans to promote participant retention and complete follow-up, including list of any outcome data to be collected for participants who discontinue or deviate from intervention protocols

For peer review only - http://bmjopen.bmj.com/site/about/guidelines.xhtml

Data management	19	Plans for data entry, coding, security, and storage, including any related processes to promote data quality (eg, double data entry; range checks for data values). Reference to where details of data management procedures can be found, if not in the protocol<br><br>All of the above – see 'Assessment schedule' and 'Outcome measures' sections
Statistical methods	20a	Statistical methods for analysing primary and secondary outcomes. Reference to where other details of the statistical analysis plan can be found, if not in the protocol
	20b	Methods for any additional analyses (eg, subgroup and adjusted analyses)
	20c	Definition of analysis population relating to protocol non-adherence (eg, as randomised analysis), and any statistical methods to handle missing data (eg, multiple imputation)<br><br>All of the above – see 'Planned analysis' section
**Methods: Monitoring**		
Data monitoring	21a	Composition of data monitoring committee (DMC); summary of its role and reporting structure; statement of whether it is independent from the sponsor and competing interests; and reference to where further details about its charter can be found, if not in the protocol. Alternatively, an explanation of why a DMC is not needed
	21b	Description of any interim analyses and stopping guidelines, including who will have access to these interim results and make the final decision to terminate the trial<br><br>See 'Trial management' section
Harms	22	Plans for collecting, assessing, reporting, and managing solicited and spontaneously reported adverse events and other unintended effects of trial interventions or trial conduct<br><br>See 'Adverse event reporting and harms' section
Auditing	23	Frequency and procedures for auditing trial conduct, if any, and whether the process will be independent from investigators and the sponsor<br><br>See 'Trial management' section
**Ethics and dissemination**		
Research ethics approval	24	Plans for seeking research ethics committee/institutional review board (REC/IRB) approval

For peer review only - http://bmjopen.bmj.com/site/about/guidelines.xhtml

Protocol amendments	25	Plans for communicating important protocol modifications (eg, changes to eligibility criteria, outcomes, analyses) to relevant parties (eg, investigators, REC/IRBs, trial participants, trial registries, journals, regulators)<br><br>For both – see 'Ethics and dissemination' and 'Abstract'
Consent or assent	26a	Who will obtain informed consent or assent from potential trial participants or authorised surrogates, and how (see Item 32)
	26b	Additional consent provisions for collection and use of participant data and biological specimens in ancillary studies, if applicable<br><br>All of the above - see 'Recruitment' and 'Consent' sections
Confidentiality	27	How personal information about potential and enrolled participants will be collected, shared, and maintained in order to protect confidentiality before, during, and after the trial<br><br>We will act to preserve participant confidentiality and will not disclose or reproduce any information by which participants could be identified, except where specific consent is obtained. Data will be stored in a secure manner (including on secure university servers) and will be registered in accordance with the General Data Protection Regulation 2018.
Declaration of interests	28	Financial and other competing interests for principal investigators for the overall trial and each study site<br><br>See 'Competing interests statement'
Access to data	29	Statement of who will have access to the final trial dataset, and disclosure of contractual agreements that limit such access for investigators<br><br>This can be provided, and is in the original protocol
Ancillary and post-trial care	30	Provisions, if any, for ancillary and post-trial care, and for compensation to those who suffer harm from trial participation<br><br>This can be provided
Dissemination policy	31a	Plans for investigators and sponsor to communicate trial results to participants, healthcare professionals, the public, and other relevant groups (eg, via publication, reporting in results databases, or other data sharing arrangements), including any publication restrictions<br><br>See 'Ethics and dissemination'

For peer review

	31b	Authorship eligibility guidelines and any intended use of professional writers<br><br>We follow the BMJ Open guidelines. No professional writers were involved.
	31c	Plans, if any, for granting public access to the full protocol, participant-level dataset, and statistical code<br><br>We are submitting the protocol for publication.
**Appendices**		
Informed consent materials	32	Model consent form and other related documentation given to participants and authorised surrogates<br><br>This can be provided
Biological specimens	33	Plans for collection, laboratory evaluation, and storage of biological specimens for genetic or molecular analysis in the current trial and for future use in ancillary studies, if applicable<br><br>N/A

*It is strongly recommended that this checklist be read in conjunction with the SPIRIT 2013 Explanation & Elaboration for important clarification on the items. Amendments to the protocol should be tracked and dated. The SPIRIT checklist is copyrighted by the SPIRIT Group under the Creative Commons "Attribution-NonCommercial-NoDerivs 3.0 Unported" license.

For peer review only

BMJ Open

Further development and feasibility randomised controlled trial of a digital programme for adolescent depression, MoodHwb: study protocol

Journal:	*BMJ Open*
Manuscript ID	bmjopen-2022-070369.R1
Article Type:	Protocol
Date Submitted by the Author:	12-Apr-2023
Complete List of Authors:	Bevan Jones, R; Cardiff University, Division of Psychological Medicine and Clinical Neurosciences; Cwm Taf Morgannwg University Health Board<br>Merry, S; The University of Auckland, Faculty of Medical and Health Sciences<br>Stallard, Paul; University of Bath, Department for Health<br>Randell, Elizabeth; Cardiff University Centre for Trials Research<br>Weavers, Bryony; Cardiff University, Division of Psychological Medicine and Clinical Neurosciences<br>Gray, Anna; Cardiff University, Division of Psychological Medicine and Clinical Neurosciences<br>Hindle, Elaine; University of Glasgow, MRC/CSO Social and Public Health Sciences Unit<br>Gavigan, Marcela; University of Glasgow, MRC/CSO Social and Public Health Sciences Unit<br>Clarkstone, Samantha; Cardiff University Centre for Trials Research<br>Williams-Thomas, Rhys; Cardiff University, Centre for Trials Research<br>Poile, Vince; Cardiff University, Centre for Trials Research<br>Playle, Rebecca; Cardiff University, Centre for Trials Research<br>Bisson, Jonathan ; Cardiff University, Division of Psychological Medicine and Clinical Neurosciences<br>McNamara, Rachel; Cardiff University, Centre for Trials Research<br>Rice, F; Cardiff University, Division of Psychological Medicine and Clinical Neurosciences<br>Simpson, Sharon; University of Glasgow, MRC/CSO Social and Public Health Sciences Unit
**Primary Subject Heading**:	Mental health
Secondary Subject Heading:	Paediatrics, Research methods
Keywords:	Child & adolescent psychiatry < PSYCHIATRY, MENTAL HEALTH, Depression & mood disorders < PSYCHIATRY, Clinical trials < THERAPEUTICS

SCHOLARONE™
Manuscripts

Further development and feasibility randomised controlled trial of a digital programme for adolescent depression, MoodHwb: study protocol

Rhys Bevan Jones[1-3], Sally Merry[4], Paul Stallard[5], Elizabeth Randell[6], Bryony Weavers[1,2], Anna Gray[1,2], Elaine Hindle[7], Marcela Gavigan[7], Samantha Clarkstone[6], Rhys Williams-Thomas[6], Vincent Poile[6], Rebecca Playle [6], Jonathan Bisson[1,2], Rachel McNamara[6], *Frances Rice[1,2], *Sharon Anne Simpson[7]

*Joint senior authors

1. Division of Psychological Medicine and Clinical Neurosciences, Cardiff University, Wales.
2. Wolfson Centre for Young People's Mental Health, Cardiff University, Wales.
3. Cwm Taf Morgannwg University Health Board, Wales.
4. Faculty of Medical and Health Sciences, University of Auckland, New Zealand.
5. Department for Health at the University of Bath, England.
6. Centre for Trials Research, Cardiff University, Wales.
7. Medical Research Council/Chief Scientist Office (MRC/CSO) Social and Public Health Sciences Unit, University of Glasgow, Scotland.

Corresponding author:

Dr Rhys Bevan Jones, Division of Psychological Medicine & Clinical Neurosciences, Cardiff University, Hadyn Ellis Building, Maindy Rd, Cardiff, Wales CF24 4HQ. Email: bevanjonesr1@cardiff.ac.uk

Word count 4263

For peer review only

Abstract

Introduction: A digital programme, MoodHwb, was co-designed with young people experiencing or at high risk of depression, parents/carers, and professionals, to provide support for young people with their mood and wellbeing. A preliminary evaluation study provided support for the programme theory and found that MoodHwb was acceptable to use. The present study aims to refine the programme based on user feedback, and to assess the acceptability and feasibility of the updated version and study methods.

Methods and analysis: Initially, this study will refine MoodHwb with the involvement of young people, including in a pre-trial acceptability phase. This will be followed by a multi-centre feasibility randomised controlled trial comparing MoodHwb plus usual care with a digital information pack plus usual care. Up to 120 young people aged 13-19 years with symptoms of depression and their parents/carers will be recruited through schools, mental health services, youth services, charities, and voluntary self-referral in Wales and Scotland. The primary outcomes are the feasibility and acceptability of the MoodHwb programme (including usage, design, and content) and of trial methods (including recruitment and retention rates), assessed two months post-randomisation. Secondary outcomes include potential impact on domains including depression knowledge and stigma, help-seeking, wellbeing and depression and anxiety symptoms measured at two months post-randomisation.

Ethics and dissemination: The pre-trial acceptability phase was approved by the Cardiff University School of Medicine Research Ethics Committee (REC) and the University of Glasgow College of Medicine, Veterinary and Life Sciences REC. The trial was approved by Wales NHS REC 3 (21/WA/0205), the Health Research Authority (HRA), Health and Care Research Wales (HCRW), university health board R&D departments in Wales, and schools in Wales and Scotland. Findings will be disseminated in peer-reviewed open-access journals, at conferences and meetings, and online to academic, clinical, and educational audiences and the wider public.

Trial registration number: ISRCTN12437531.

Strengths and limitations of this study

- MoodHwb was co-designed with young people with lived experience or at high risk of depression, parents/carers, and practitioners.

For peer review only

- MoodHwb is bilingual, personalised according to user needs and preferences, and has been developed in line with evidence-based approaches to support young people with depressive symptoms and their parents/carers, friends, and practitioners.
- Multiple methods will be used to collect data in a range of settings for the trial, including validated questionnaires, semi-structured interviews, a focus group, and Web/app usage monitoring.
- As this is a feasibility study, we will not be able to determine the effectiveness of MoodHwb; however, the findings will inform the design of a definitive effectiveness trial.
- Participants will not be blind to treatment allocation as this is not possible with an intervention of this kind.

For peer review only

BACKGROUND

Depression is common in adolescence, and is associated with social and educational impairments, deliberate self-harm, and suicide. It can also mark the beginning of long-term mental health difficulties. Subthreshold depressive symptoms can also affect quality of life and are a risk indicator for depressive disorder. Early treatment and prevention of adolescent depression is therefore a major public health concern[1,2]. There are also concerns about the economic burden of mental disorders[3,4]. However, most adolescents with depression do not access or receive formal help and engaging young people (YP) in prevention and early intervention programmes is challenging[5-7].

Guidelines for the prevention and management of depression in YP (e.g., National Institute for Health and Care Excellence (NICE)[8]; American Academy of Child & Adolescent Psychiatry (AACAP)[9]) stress the need for good information and evidence-based psychosocial interventions for YP and families/carers, and digital interventions are recommended for mild depression in UK NICE guidelines[8]. Digital mental health technologies (i.e., resources and interventions) are a potential way of increasing access to support, at relatively low cost. Most YP in high-income countries have access to the internet and mobile devices, including in the UK[10,11]. Many in low and middle-income countries (LMICs) can also access the internet, where there are few alternative sources of support[12]. Whilst there is increasing evidence for the effectiveness of technologies for adolescent depression[13,14], more programmes are needed to support adolescents that are accessible, evidence-based, developed with user input and evaluated in line with research frameworks – and to provide greater options for YP in accessing help[15].

MoodHwb is a bilingual (English and Welsh), interactive, and multi-platform digital programme

designed to support YP with their mood and wellbeing - and their families/carers, friends, and practitioners (**figure 1**).

FIGURE 1 HERE

It was co-designed using a series of interviews and focus groups with YP experiencing or at high risk of depressive symptoms (due to family history), parents/carers, and professionals, alongside workshops with a digital media company and an animator[16-18]. The development was also informed by a systematic review[19], design, educational and psychological theories, and a preliminary 'logic model' (programme theory)(**figure 2**).

FIGURE 2 HERE

As part of the developmental work, there was an early evaluation of MoodHwb with a small number of YP and their parents/carers and practitioners, recruited via primary and secondary Child and Adolescent Mental Health Services (CAMHS), school counsellors and nurses, and a previous study sample (Early Prediction of Adolescent Depression (EPAD) study)[20]. Preliminary results were encouraging, and participants found the programme acceptable to use and gave feedback on how it could be improved. Findings also provided initial support for the programme theory, particularly regarding depression literacy, self-efficacy, and depressive symptoms. In line with guidance for complex interventions, a larger feasibility trial is now required[21,22].

The primary aims of the current study are to further refine and evaluate the feasibility, acceptability, and potential impact of the digital programme MoodHwb and the feasibility and acceptability of the trial methods.

METHODS AND ANALYSIS

Initial development phase

The first phase involves the further refinement of the digital programme, based on the feedback provided by participants during the earlier evaluation. The key revisions recommended included improving the navigation, audiovisual and interactive elements, making it more personalised based

For peer review only

on user preferences, developing the app version, and expanding the self-help and anxiety content[20]. There were subsequent consultations with practitioners at the proposed trial recruitment centres across Wales and Scotland, and researchers in the field.

Regular updates are required to the design and content of digital programmes because of the fast-moving pace of the field and the emerging evidence and guidelines. We have completed reviews of the co-design of digital technologies with children and YP[18] and technologies to support YP with depression and anxiety[15]. The principal investigator (PI) has visited research centres in youth and digital mental health in New Zealand, Australia, and the Republic of Georgia. Workshops and meetings will be held with the digital media company and animator that helped to develop the original prototype.

As part of a pre-trial acceptability phase, YP in Wales and Scotland (n=10-15) will be given access to the new prototype. They will be recruited through school counsellors, charities, youth advisory groups (YAGs) and self-referrals. Adolescents will be eligible to take part if they are aged 13-19 years and have sufficient understanding of the English language, access to the internet and a valid email address. They will be interviewed to ensure that the changes made are acceptable ahead of the trial, and to explore issues of transferability in Scotland, given that the programme was developed in Wales. We will aim to recruit some YP who have experienced depressive symptoms. However, others with no experience of mental health difficulties will also give feedback e.g., on whether the programme is user-friendly and age-appropriate. Practitioners from health, education and youth services will be consulted further regarding the new prototype through meetings and online communication.

For peer review only

Trial design

The feasibility study is a multi-centre feasibility randomised controlled trial (RCT) comparing the MoodHwb programme plus usual care (if the young person is receiving support) to digital information sheets on mood and wellbeing plus usual care (if receiving support)(**figure 3**). The research plan follows guidance for complex interventions[21,22], and digital person-centred interventions[23].

FIGURE 3 HERE

Setting and participants

Trial participants will be identified by practitioners in schools (by e.g., counsellors, nurses, teachers), mental health centres (mainly primary services), charities, youth services and sports academies in Wales, and in schools and charities via the second site at the University of Glasgow, Scotland. The School Health Research Network (SHRN, Wales) and Schools Health and Wellbeing Improvement Research Network (SHINE, Scotland) will be involved. These sources were chosen as the developmental work suggested that the most appropriate setting for the programme would be the time when depressive difficulties start and YP first present - and is therefore a form of early intervention[17,20]. We will also recruit self-referrals, for example through social media and online advertising, including through the National Centre for Mental Health (NCMH), Wolfson Centre for YP's Mental Health (WCYPMH) and TRIUMPH youth mental health network.

Young people will be eligible if they: (1) are aged 13-19 years, (2) are experiencing depressive symptoms, (3) have a sufficient understanding of English to complete outcomes measures, (4) have access to the internet and a valid email address, and (5) live in Wales or Scotland. Exclusion criteria are: (1) already receiving specialist treatment for depression (e.g., cognitive behavioural therapy (CBT) in secondary CAMHS), and (2) the presence of other mental health difficulties where alternative support might be appropriate. Parents and carers will be targeted for the trial if they have an adolescent with depressive symptoms. Professionals will be approached if they work with YP who present with mental health difficulties.

For peer review only

Sample size

We aim to recruit up to 120 adolescent participants, and their parents/carers. Approximately two-thirds of the participants will be from Wales and one-third from Scotland. We expect a dropout of around 30%, based on previous digital health studies and the development and early evaluation work. With a sample size of 120 we can estimate the overall expected retention rate of 0.7 to within +/-0.082 (70% +/-8.2%) and within group retention as 0.7+/-0.116 based on the width of a two-sided 95% confidence interval (CI).

Recruitment

School, mental health, youth, or charity practitioners will identify potentially eligible YP and ask them whether they are interested in participating. The practitioners will provide a study postcard which directs the YP to the bilingual study website (www.ncmh.info/digital-support-study/), where there will be details of the study and information sheets. We will ask practitioners to keep a screening log of all YP approached. There will be a link to the website via social media and online advertisements for self-referrals.

If they are interested in taking part, YP will be asked to complete an online eligibility form on the website. This will include questions on their contact details and to assess whether they meet the eligibility criteria, including items from the Mood and Feelings Questionnaire (MFQ)[24] to measure depressive symptoms. They would be eligible if they score 20 or over on the MFQ. If they score 29 or over, they would also be advised to seek help and we will contact their general practitioner (GP), as this indicates a 'major depressive disorder'[25]. They will not be eligible if they access specialist treatment (e.g. CBT; see exclusion criteria). The MFQ is commonly used in clinical and research practice and is a recommended screening tool in NICE guidelines for depression in children and young people[8]. The questionnaire and these cut-offs have also been used in previous trials of digital interventions for depression in YP[26,27]. The research team will then contact those who are interested to discuss the project (including consent forms and questionnaires), confirm their eligibility, respond to their questions, and ask whether their parents/carers are interested in participating.

Consent

Online consent forms are provided for the participants to complete, before completion of baseline questionnaires. If under 16 years old, YP will be asked to provide assent while their parent or legal guardian will be asked for consent. Dual consent and assent will be required for participation. Those aged 16 years or older will be able to provide their own consent. Parents/guardians and professionals who participate will also be asked to complete consent forms.

Randomisation

For peer review only

Participants will be randomised at a 2:1 ratio to either the digital programme, MoodHwb plus usual care, or digital information sheets on mood and wellbeing in YP plus usual care (**figure 3**). We decided to randomise at a 2:1 ratio to allow for more information to be gained about those receiving the intervention. The permuted block randomisation will be computerised and stratified according to site (Wales and Scotland). This will be built into the study database overseen by the Centre for Trials Research (CTR, Cardiff University), providing remote access and preserving allocation concealment. Because of the nature of the intervention, participants will not be blind to their allocation, however data analysis will be carried out blind to allocation.

Intervention

MoodHwb is a digital programme to support YP with their mood and wellbeing, particularly those with elevated depressive symptoms. It can also be used by their families/carers, friends, and practitioners. The programme and development process are described in detail in previous publications[16-18].

It is designed to engage YP by using developmentally appropriate language, illustrations, animations, and interactive components. It aims to promote self-help, help-seeking where appropriate and social support. Whilst it was founded primarily on psychoeducation, it also includes elements of CBT, behavioural activation, positive psychology, and interpersonal, family systems and behaviour change theories. The first version was developed primarily as a website, although the interactive sections (e.g., mood diary) are also available in an 'app'. It could be used independently as a 'standalone' programme by YP (or parents/carers, friends, or professionals) or supported by another person.

From the welcome screen, the user is asked questions (e.g., regarding their mood and anxiety levels), and the answers (i) are stored in their 'profile' section, and (ii) help to signpost to the relevant subsections on the subsequent 'dashboard' screen, therefore personalising the content. Along with this 'mood diary', there are other interactive elements: a goal-setting element and a section to save links to helpful resources. Five sections were developed for YP (**figure 1**), and these cover: (i) low mood and depression, (ii) possible reasons, (iii) self-help approaches, (iv) sources of help and (v) associated health difficulties. There is a sixth section to support those who might be concerned about YP, such as parents/carers, friends, and practitioners. It is recommended that users navigate sections in this order, but they can use it in any way that is helpful for them. Each of these sections includes information, animations, personal stories, exercises, and links to relevant resources. MoodHwb is bilingual - 'hwb' is the Welsh translation for 'hub', and can mean a 'lift' or

For peer review only

'boost'. HwbHwyliau is the Welsh name for the programme.

Participants in the control arm will be emailed a digital information pack, which will consist of digital information sheets on mood and wellbeing (including depression) from the NCMH, Royal College of Psychiatrists and YoungMinds websites. Such resources offer general information, are widely accessible online, and have been used by control arm participants in similar trials [e.g., 27].

Patient and public involvement

The programme was co-designed using a series of interviews (n=12) and focus groups (n=6) with (i) YP who either had a history of depression or were at elevated risk, (ii) parents/carers, and (iii) professionals. Participants were recruited from CAMHS in Wales, and families from the EPAD study. As noted, further feedback on the programme was provided during the early evaluation[20].

The project has been adopted by the Centre for Development, Evaluation, Complexity and Implementation in Public Health Improvement (DECIPHer, Cardiff University), and the study plans were developed in collaboration with their YAG, ALPHA (Advice Leading to Public Health Advancement). We will collaborate further with them as well as the YAGs of the WCYPMH, NCMH and TRIUMPH network. A young person has been invited to join the Trial Steering Committee (TSC), and other YP (aged 13 to 16 years) have reviewed study documents e.g., information sheets and consent forms. Practitioners and researchers have been consulted regarding the plans.

Assessment schedule

Data will be collected at (i) baseline and (ii) follow-up, two months after randomisation. Data will be collected from (i) baseline and follow-up online questionnaires; (ii) semi-structured interviews with YP and parents/carers who used the programme; (iii) a focus group with professionals and (iv) Web/app usage data (**table 1**).

For peer review only

Table 1: Schedule of enrolment, interventions and assessments for the trial[1]

Time point / Activity	Screening (contact 1)	Baseline (contact 1) Inter-vention	Baseline (contact 1) Control	Month 2 (contact 2) Inter-vention	Month 2 (contact 2) Control
**Referral to the trial/Register of interest**	X				
**Eligibility assessment**	X				
**Consent process**	X				
**Randomisation**	X				
**Access to MoodHwb**		X			
**Questionnaires**					
Adolescent Depression Knowledge Questionnaire (ADKQ)[28]		•/○	•/○	•/○	•/○
Depression Stigma Scale (DSS)[29]		•/○	•/○	•/○	•/○
General Self-Efficacy Scale (GSE)[30]		•	•	•	•
General Health-Seeking Questionnaire (GHSQ)[31]		•	•	•	•
Warwick-Edinburgh Mental Wellbeing Scales (WEMWBS)[32]		•/○	•/○	•/○	•/○
Mood and Feelings Questionnaire (MFQ)[24]		•/○	•/○	•/○	•/○
Revised Children's Anxiety and Depression Scale-Short Version (RCADS-25)[33]		•/○	•/○	•/○	•/○
Hospital Anxiety and Depression Scale (HADS)[34]		○	○	○	○
Items on Health economics & Life Events[35]		•/○	•/○	•/○	•/○
Items on use and acceptability of MoodHwb				•/○	
**Process evaluation**					
Young person interview (n=20-25)				•	
Parent/carer Interview (n=10-15)				○	
Professionals focus group				*	

(• participant/○ parent/*professionals) 'Contact' will comprise phone/videoconferencing discussion and completion of online forms/questionnaires. Taken from the HRA CTIMP protocol template (2016).

For peer review only - http://bmjopen.bmj.com/site/about/guidelines.xhtml

We will use remote methods for recruitment, consent, and data collection (e.g., phone, email, videoconferencing, online forms and questionnaires) because these have been shown to be efficient and effective ways of conducting research during the Covid-19 pandemic. This approach is consistent with the digital nature of the programme and would enable us to reach more YP. All participants will be offered £10 gift vouchers after (i) completing baseline questionnaires, (ii) completing follow-up questionnaires, and (iii) participating in interviews.

Baseline

At baseline, YP in both arms will complete questionnaires which will include items on their demographic and contact information, health and use of services, and standardised questionnaires on knowledge, stigma, self-efficacy, help-seeking, wellbeing, symptoms, and life stresses (**table 1**). The parent/carer questionnaires will include items on the young person's details, use of services and symptoms, and their knowledge, beliefs, wellbeing, and symptoms. All participants will also complete items on the acceptability of the questionnaires.

Follow-up

All baseline measures will be repeated at the follow-up timepoint, two months post-randomisation. In addition, those in the intervention arm will be asked about their use of the programme, and their views on the content and design. Those in the control arm will be asked whether they had access to MoodHwb, to explore contamination. A subgroup of YP (n=up to 20-25) and parents/carers (n=up to 10-15) who used the programme will be interviewed at follow-up on their use of and views of MoodHwb and the evaluation process. The Web/app usage of those using the programme will be monitored using Google Analytics up to 6 months post-randomisation, to provide information on longer-term use. There will be a focus group for professionals who work with YP from a range of settings, to ascertain their views.

For peer review only

Process evaluation

The interviews with YP and parents/carers at follow-up and practitioner focus group noted earlier will provide more in-depth information on the acceptability and feasibility of the programme and evaluation process – in addition to the questionnaire and Web/app usage data. Participants will be purposively selected for interview based on their usage of MoodHwb (i.e., frequent or occasional), age, gender, support received (e.g. school, health service), and primary language (English, Welsh) – so that there is a broad range of viewpoints. A process evaluation framework has been developed, following MRC guidance[36], and focuses primarily on the intervention but also examines certain trial processes. It addresses issues regarding reach, contamination, retention, recruitment, context, exposure, and fidelity.

Outcome measures

Primary outcome

The feasibility and acceptability of MoodHwb outcomes will include the level of usage and adherence (including number of times accessed and duration of use), views and acceptability of the design and content (including strengths, areas to develop further and approaches to integrate into services and charities), and technical and accessibility aspects. The feasibility and acceptability outcomes of the trial methods will include recruitment and retention rates, completeness of measures, representativeness, randomisation, contamination, and acceptability (particularly of the remote methods). Progression criteria for a full trial have been developed with the TSC, based on these outcomes (**appendix 1**).

Secondary outcomes

Secondary outcomes assess the potential impact of the programme on a core set of domains, including depression literacy or knowledge, depression stigma, help-seeking behaviour, self-efficacy, wellbeing, and depression and anxiety symptoms. These will be measured via standardised validated questionnaires (**table 1**), and participant feedback (via questionnaires and interviews).

For peer review only

Exploratory economic evaluation

We will investigate the feasibility and acceptability of collecting health economic data. We will identify and measure the resources involved in intervention delivery as well as resource use by participants, with reference to methodology from health economics guidance[37,38]. An estimation of the following will be collected: i) intervention costs (development, hosting, and maintenance costs), ii) health (e.g., contact with GPs, hospitals and other health settings, medication), social, educational, and personal resource costs, using questionnaire items adapted from the Client Service Receipt Inventory, iii) wellbeing, using WEMWBS[32].

Planned analysis

Primary outcome findings will be derived primarily from analysis of the questionnaires, interviews, and focus group, with additional analysis of Web/app usage data regarding usage, adherence and technical aspects, and screening logs and participant database regarding recruitment and retention. Secondary outcome findings will be derived primarily from analysis of validated questionnaires.

Participant and Web usage data

The flow of participants through the trial will be presented as a CONSORT flowchart[39]. Baseline demographic data will be presented for all consented and randomised participants. This will be summarised and tabulated for the whole group and by arm, using mean (SD) or median (IQR) where appropriate. All subsequent summaries will use complete cases and will employ the intention to treat principle (participants will remain in groups to which they were randomised irrespective of intervention received). Web usage data from Google Analytics will be tabulated, for example related to number of times accessed, duration of use, sections accessed, and language.

Qualitative analysis

For peer review only

The interviews and focus group recordings will be transcribed, and data analysis will proceed using a thematic analysis approach. This is a process of identifying, analysing, reporting, and interpreting patterns or themes, and is one of the most used qualitative approaches in health research[40]. Codes will be applied to broad themes and then broken down further into sub-codes - 30% will be double-coded to ensure the reliability of coding, and differences will be resolved through discussion. Transcripts will be closely examined to identify key themes and sub-themes. Thematic analysis will be supported by the computer-assisted qualitative analysis software, NVivo.

Quantitative analysis

Baseline questionnaire data will be scored according to the validated scoring algorithms from the relevant literature. Missing item-by-item data will be handled according to the scoring algorithm for each measure individually. Baseline scores will be presented descriptively, using summary statistics (means, SD, medians, IQR and percentages with associated 95% CI where appropriate) for the whole group and by arm. Distributional assumptions will be checked, and data transformation carried out if required. A comparison of randomised groups will be tabulated but not tested, in line with the feasibility aims. A comparison of baseline data for those completing and not completing the study will be tabulated to assess drop-out bias.

Scores at the two-month time point will be tabulated and compared with baseline in a complete case analysis. No hypothesis tests will be carried out but 95% CI for the difference at two months between the two arms will be estimated and reported. These will be adjusted for baseline score and any factors such as site used to balance the randomisation. Statistical analysis will be performed using Stata software, but no p-values will be presented. We will further develop and test the MoodHwb logic model (**figure 2**) using multiple methods, and examine possible mechanisms of action and active components. This will help to define a core set of outcome measures (related to the model) and identify the most appropriate primary outcome for evaluation in an effectiveness trial.

Trial management

A Trial Management Group (TMG) and an independent TSC have been established to monitor progress and advise the researchers. The TMG will meet approximately every 8-10 weeks during the

For peer review only

trial planning and throughout the trial. The TMG is composed of the PI, co-investigators, research assistant, trial administrator, CTR members, and University of Glasgow site team. The TSC will meet approximately every 12 months. The TSC includes a young person, and an independent digital mental health researcher, statistician, and mixed-methods researcher.

Adverse event reporting and harms

For this trial, adverse events (AEs) are defined as any untoward medical occurrence in a participant administered an intervention and which are not necessarily caused by or related to that intervention. This would include significant deterioration in mental health symptoms, including depressive and anxiety symptoms, and emergence of thoughts of self-harm. Serious adverse events (SAEs) include those resulting in death, are life-threatening, require hospitalisation or prolongation of existing hospitalisation, result in persistent or significant disability or incapacity, or other medically important conditions. The PI will assess each SAE for expectedness. A standard study/departmental procedure for documenting and dealing appropriately with these events will be followed. Young people and parents/carers will be signposted to appropriate resources and services if there are concerns at the screening stage (see recruitment section) or from the questionnaire or interview responses if recruited. Their GP details will be collected at the screening and baseline stages, so they can be contacted. This is especially important, as the study will recruit self-referrals who might not access other support. An urgent safety measure is an action that the Sponsor or PI may carry out to protect the participants of a trial against any immediate hazard to their health or safety – and must be notified to the REC immediately.

For peer review only

Ethics and dissemination

Approvals were received from the Cardiff University School of Medicine REC and University of Glasgow College of Medical, Veterinary and Life Sciences REC for the pre-trial acceptability phase with YP. Favourable ethical opinion for the overall study and trial was obtained from Wales REC 3 (reference 21/WA/0205), and approvals were received by HRA, HCRW, university health board R&D departments in Wales, and schools in Wales (including via SHRN) and Scotland (including via SHINE). The study was registered with ISRCTN (ISRCTN12437531). Approvals were received for MoodHwb to be added to the (Apple) App Store and Google Play App Store.

We will disseminate our findings to academics, researchers, policymakers, and practitioners through high-impact open-access publications and presentations at relevant academic, clinical, and educational conferences and meetings. The findings will be disseminated online, for example on the NCMH, WCYPMH and TRIUMPH websites. Results will be made available to all participants after the study completion.

DISCUSSION

MoodHwb is a digital programme to support YP with their mood and wellbeing, especially those experiencing elevated depressive symptoms, and families/carers, friends, and professionals who are involved in supporting them. It can be used as a website and as an app, as a standalone programme or supported by others, for example in counselling or therapy sessions. Digital mental health technologies such as this have the potential to improve reach and access to therapies at a relatively low cost. MoodHwb will help to address the need for accessible, evidence-based technologies that are developed with user input and evaluated rigorously – and will provide YP and others with more options when looking for support.

Strengths and limitations

MoodHwb was co-designed with YP with lived experience or at high risk of depression, parents/carers, and practitioners. The programme is bilingual, interactive, personalised according to user needs and preferences, and multi-platform. It has been developed in line with evidence-based approaches, not only for YP but also for those concerned about them. Multiple methods will be used to collect data in a range of settings, including validated questionnaires, semi-structured interviews, a focus group, and Web/app usage monitoring. The involvement of participants in the second site in Scotland will help to boost recruitment and enable the exploration of transferability.

Given that this is a feasibility study, we will not be able to explore effectiveness; however the information and experience will inform the design of a future effectiveness trial. Whilst it is not feasible to blind participants to allocation; data analysis will be carried out blind to allocation. We have minimised the risk of contamination between trial arms by ensuring that a log-in is required to access MoodHwb. There will be items in the follow-up questionnaire for participants in the control arm on whether they have accessed MoodHwb during the study.

For peer review only

Having access to the internet and an email address is one of the inclusion criteria for the trial, however, some participants might not be adept at using digital devices (e.g. mobiles, desktop computers), or might prefer other approaches (e.g. face-to-face, printed). This might affect adherence to the digital programme and retention in the study, and we will explore methods of engaging participants during the study, for example via questionnaires and interviews.

At the end of the study, there will be a period of reflection and planning regarding what further development, evaluation and implementation work are required for MoodHwb, both as a standalone and supported technology, in collaboration with YP. If it is ultimately found to be feasible and effective, it could be rolled out to help YP, families/carers, and practitioners across a range of settings.

Trial status

Recruitment started in November 2022 and will end in August 2023.

Trial sponsor

Cardiff University is the Sponsor of the study.

Authors' contributions

RBJ designed and obtained funding for the study, with research support from SAS, FR, SM, PS and JB, and advice from ER, RM, RP, SC, VP, and RWT (CTR). BW and AG provided research assistance at Cardiff University, and EH and MG coordinated the study at the University of Glasgow. RBJ drafted the initial study protocol. All authors contributed to and approved the final version.

Funding statement

This work was supported by a National Institute for Health Research (NIHR) and Health and Care Research Wales (HCRW) Post Doctoral Fellowship programme, grant number NIHR-PDF-2018, awarded to RBJ. SS was supported by UK MRC and Scottish Chief Scientist Office core funding as part of the MRC/CSO Social and Public Health Sciences Unit 'Complexity in Health Improvement' programme (MC_UU_12017/14 and SPHSU14) and MC-PC_13027. SM was supported by Cure Kids,

For peer review only

New Zealand. The work was also supported by the NCMH, WCYPMH (established with support from the Wolfson Foundation) and TRIUMPH network.

Acknowledgements

The authors would like to thank all the YP, parents, carers, professionals, and researchers who participated in the development of this protocol. We would like to thank Caroline Warren, Gareth Watson, and Tim Pickles (all Cardiff University) for their contribution to the trial administration, database and statistics respectively, and Emma McIntosh and Kathleen Boyd (both University of Glasgow) for their contributions to the health economics. We thank the TSC for their support.

Competing interests statement

The authors declare they have no competing or potential conflicts of interest.

Figure legend/captions

Figure 1: MoodHwb (v1) welcome screen (main image/left) and open menu (right) (from Bevan Jones et al. 2020a)

Figure 2: Logic model for MoodHwb (above, from Bevan Jones et al 2018b), including potential pathways (below)

Figure 3: Participant flow diagram

Table 1: Schedule of enrolment, interventions and assessments for the trial

Supplementary material:

Proposed progression criteria

Trial protocol

Consent forms for young person

For peer review only

REFERENCES

1. Thapar, A., Eyre, O., Patel, V., Brent, D., 2022. Depression in young people. Lancet 400, 617–631. https://doi.org/https://doi.org/10.1016/S0140-6736(22)01012-1

2. Bertha EA, Balázs J. Subthreshold depression in adolescence: a systematic review. Eur Child Adolesc Psychiatry. 2013 Oct;22(10):589-603. doi: 10.1007/s00787-013-0411-0

3. Knapp, M., & Wong, G. (2020). Economics and mental health: the current scenario. *World Psychiatry : Official Journal of the World Psychiatric Association (WPA)*, *19*(1), 3–14. https://doi.org/10.1002/WPS.20692

4. Trautmann, S., Rehm, J., & Wittchen, H. (2016). The economic costs of mental disorders: Do our societies react appropriately to the burden of mental disorders? *EMBO Reports*, *17*(9), 1245. https://doi.org/10.15252/EMBR.201642951

5. Potter, R., Mars, B., Eyre, O., Legge, S., Ford, T., Sellers, R., … Thapar, A. K. (2012). Missed opportunities: mental disorder in children of parents with depression. *The British Journal of General Practice : The Journal of the Royal College of General Practitioners*, *62*(600), e487-93. https://doi.org/10.3399/bjgp12X652355

6. Neufeld, S. A. S., Dunn, V. J., Jones, P. B., Croudace, T. J., & Goodyer, I. M. (2017). Reduction in adolescent depression after contact with mental health services: a longitudinal cohort study in the UK. *The Lancet. Psychiatry*, *4*(2), 120–127. https://doi.org/10.1016/S2215-0366(17)30002-0

7. Bennett, C., Jones, R. B., & Smith, D. (2014). Prevention strategies for adolescent depression. *Advances in Psychiatric Treatment*, *20*(2), 116–124. https://doi.org/10.1192/apt.bp.112.010314

8. National Institute for Health and Clinical Excellence (NICE). (2019). Depression In Children and Young People. *NICE Clinical Guideline*. Retrieved from http://guidance.nice.org.uk/CG28/Guidance

9. Birmaher, B., Brent, D., 2007. Practice Parameter for the Assessment and Treatment of Children and Adolescents With Depressive Disorders. J. Am. Acad. Child Adolesc. Psychiatry 46, 1503–1526. https://doi.org/10.1097/CHI.0B013E318145AE1C

10. Office for National Statistics. (2019). Exploring the UK's digital divide - Office for National Statistics. *Office for National Statistics*, 1–24.

For peer review only

11. Ofcom. (2021). Online Nation 2021, (June), 16. Retrieved from https://www.ofcom.org.uk/__data/assets/pdf_file/0013/220414/online-nation-2021-report.pdf

12. Naslund, J. A., Aschbrenner, K. A., Araya, R., Marsch, L. A., Unützer, J., Patel, V., & Bartels, S. J. (2017). Digital technology for treating and preventing mental disorders in low-income and middle-income countries: a narrative review of the literature. *The Lancet Psychiatry*, *4*(6), 486–500. https://doi.org/10.1016/S2215-0366(17)30096-2

13. Hollis, C., Falconer, C. J., Martin, J. L., Whittington, C., Stockton, S., Glazebrook, C., & Davies, E. B. (2017). Annual Research Review: Digital health interventions for children and young people with mental health problems-a systematic and meta-review. 58(4): 474-503. https://doi.org/10.1111/jcpp.12663

14. Grist, R., Croker, A., Denne, M., & Stallard, P. (2019). Technology Delivered Interventions for Depression and Anxiety in Children and Adolescents: A Systematic Review and Meta-analysis. *Clinical Child and Family Psychology Review*, *22*(2), 147–171. https://doi.org/https://dx.doi.org/10.1007/s10567-018-0271-8

15. Bevan Jones, R., Hussain, F., Agha, S. S., Weavers, B., Lucassen, M., Merry, S., … Rice, F. (2022). Digital technologies to support adolescents with depression and anxiety: review. *BJPsych Advances*, 1–15. https://doi.org/DOI: 10.1192/bja.2022.3

16. Bevan Jones, R., Thomas, J., Lewis, J., Read, S., All, I. J.-R. for, & 2017, U. (2017). Translation: from bench to brain–using the visual arts and metaphors to engage and educate. *Scienceopen.Com*, *1*(2), 265–283. https://doi.org/10.18546/RFA.01.2.04

17. Bevan Jones, R., Thapar, A., Rice, F., Beeching, H., Cichosz, R., Mars, B., … Simpson, S. A. (2018b). A web-based psychoeducational intervention for adolescent depression: design and development of MoodHwb. *JMIR Mental Health*, *5*(2). https://doi.org/10.2196/mental.8894

18. Bevan Jones, R., Stallard, P., Agha, S. S., Rice, S., Werner-Seidler, A., Stasiak, K., … Merry, S. (2020b). Practitioner review: Co-design of digital mental health technologies with children and young people. *Journal of Child Psychology and Psychiatry*, *61*(8), 928–940. https://doi.org/10.1111/JCPP.13258

19. Bevan Jones, R., Thapar, A., Stone, Z., Thapar, A., Jones, I., Smith, D., & Simpson, S. (2018a). Psychoeducational interventions in adolescent depression: A systematic review. *Patient Education and Counseling*, *101*(5), 804–816. https://doi.org/10.1016/J.PEC.2017.10.015

20. Bevan Jones, R., Thapar, A., Rice, F., Mars, B., Agha, S. S., Smith, D., … Simpson, S. A. (2020a). A Digital Intervention for Adolescent Depression (MoodHwb): Mixed Methods Feasibility Evaluation. *JMIR Ment Health 2020;7(7):E14536 Https://Mental.Jmir.Org/2020/7/E14536*, *7*(7), e14536. https://doi.org/10.2196/14536

For peer review only

21. Craig, P., Dieppe, P., Macintyre, S., Mitchie, S., Nazareth, I., & Petticrew, M. (2008). Developing and evaluating complex interventions: the new Medical Research Council guidance. *BMJ*, *337*(7676), 979–983. https://doi.org/10.1136/BMJ.A1655

22. Skivington, K., Matthews, L., Simpson, S.A., Craig, P., Baird, J., Blazeby, J.M., Boyd, K.A., Craig, N., French, D.P., McIntosh, E., Petticrew, M., Rycroft-Malone, J., White, M., Moore, L., 2021. A new framework for developing and evaluating complex interventions: update of Medical Research Council guidance. BMJ 374. https://doi.org/10.1136/bmj.n2061

23. Yardley, L., Morrison, L., Bradbury, K., & Muller, I. (2015). The Person-Based Approach to Intervention Development: Application to Digital Health-Related Behavior Change Interventions. *J Med Internet Res 2015;17(1):E30 Https://Www.Jmir.Org/2015/1/E30*, *17*(1), e4055. https://doi.org/10.2196/JMIR.4055

24. Angold, A., Costello, E., Messer SC, & Pickles A. (1995). Development of a short questionnaire for use in epidemiological studies of depression in children and adolescents. *International Journal of Methods in Psychiatric Research*. Retrieved from https://psycnet.apa.org/record/1996-02633-002

25. Daviss, W., Birmaher, B., Melhem, N. A., Axelson, D. A., Michaels, S. M., & Brent, D. A. (2006). Criterion validity of the Mood and Feelings Questionnaire for depressive episodes in clinic and non-clinic subjects. *Journal of Child Psychology and Psychiatry and Allied Disciplines*, *47*(9), 927–934. https://doi.org/10.1111/j.1469-7610.2006.01646.x

26. Smith, P., Scott, R., Eshkevari, E., Jatta, F., Leigh, E., Harris, V., … Yule, W. (2015). Computerised CBT for depressed adolescents: Randomised controlled trial. *Behaviour Research and Therapy*, *73*, 104–110. https://doi.org/10.1016/J.BRAT.2015.07.009

27. Wright, B., Tindall, L., Hargate, R., Allgar, V., Trépel, D., & Ali, S. (2019). Computerised cognitive-behavioural therapy for depression in adolescents: 12-month outcomes of a UK randomised controlled trial pilot study. *BJPsych Open*, *6*(1). https://doi.org/10.1192/BJO.2019.91

28. Hart, S. R., Kastelic, E. A., Wilcox, H. C., Mary, •, Beaudry, B., Musci, R. J., … Swartz, K. L. (2014). Achieving Depression Literacy: The Adolescent Depression Knowledge Questionnaire (ADKQ). https://doi.org/10.1007/s12310-014-9120-1

29. Griffiths, K. M., Christensen, H., Jorm, A. F., Evans, K., & Groves, C. (2004). Effect of web-based depression literacy and cognitive-behavioural therapy interventions on stigmatising attitudes to depression: randomised controlled trial. *The British Journal of Psychiatry : The Journal of Mental Science*, *185*(OCT.), 342–349. https://doi.org/10.1192/BJP.185.4.342

30. Schwarzer, R., & Jerusalem, M. (1995). General Self-Efficacy Scale. *J. Weinman, S. Wright, & M. Johnston, Measures in Health Psychology: A User's Profile. Causal and Control Beliefs*, *35*. Retrieved from https://psycnet.apa.org/doiLanding?doi=10.1037%2Ft00393-000

For peer review only

31. Wilson, C. J., Deane, F. P., Ciarrochi, J., & Rickwood, D. (2005). Measuring Help-Seeking Intentions: Properties of the General Help-Seeking Questionnaire. *Canadian Journal of Counselling*, *39*(1), 15–28. Retrieved from https://psycnet.apa.org/record/2005-05989-002

32. Tennant, R., Hiller, L., Fishwick, R., Platt, S., Joseph, S., Weich, S., … Stewart-Brown, S. (2007). The Warwick-Edinburgh mental well-being scale (WEMWBS): Development and UK validation. *Health and Quality of Life Outcomes*, *5*(1), 1–13. https://doi.org/10.1186/1477-7525-5-63/TABLES/4

33. Ebesutani, C., Korathu-Larson, P., Nakamura, B. J., Higa-McMillan, C., & Chorpita, B. (2017). The Revised Child Anxiety and Depression Scale 25–Parent Version: Scale Development and Validation in a School-Based and Clinical Sample. *Assessment*, *24*(6), 712–728. https://doi.org/10.1177/1073191115627012

34. Zigmond, A. S., & Snaith, R. P. (1983). The Hospital Anxiety and Depression Scale. *Acta Psychiatrica Scandinavica*, *67*(6), 361–370. https://doi.org/10.1111/J.1600-0447.1983.TB09716.X

35. Johnson, J. H., & McCutcheon, S. M. (1980). Assessing life stress in older children and adolescents: Preliminary findings with the Life Events Checklist. *Stress and anxiety*, *7*, 111-125.

36. Moore, G. F., Audrey, S., Barker, M., Bond, L., Bonell, C., Hardeman, W., … Baird, J. (2015). Process evaluation of complex interventions: Medical Research Council guidance. *BMJ (Clinical Research Ed.)*, *350*, h1258. https://doi.org/10.1136/bmj.h1258

37. National Institute for Health and Clinical Excellence (NICE). (2012). 6 Incorporating health economics | Methods for the development of NICE public health guidance (third edition) | Guidance | NICE. Retrieved March 1, 2022, from https://www.nice.org.uk/process/pmg4/chapter/incorporating-health-economics

38. Tudor Edwards, R., & McIntosh, E. (2019). *Applied Health Economics for Public Health Practice and Research* (1st ed.). Oxford, UK: Oxford University Press. https://doi.org/10.1093/med/9780198737483.001.0001

39. Rennie, D. (2001). CONSORT revised--improving the reporting of randomized trials. JAMA 285, 2006–2007. https://doi.org/10.1001/JAMA.285.15.2006

40. Pope, C., & Mays, N. (2006). *Qualitative methods in health research* (Vol. 5). Blackwell Publishing: BMJ Books.

For peer review only

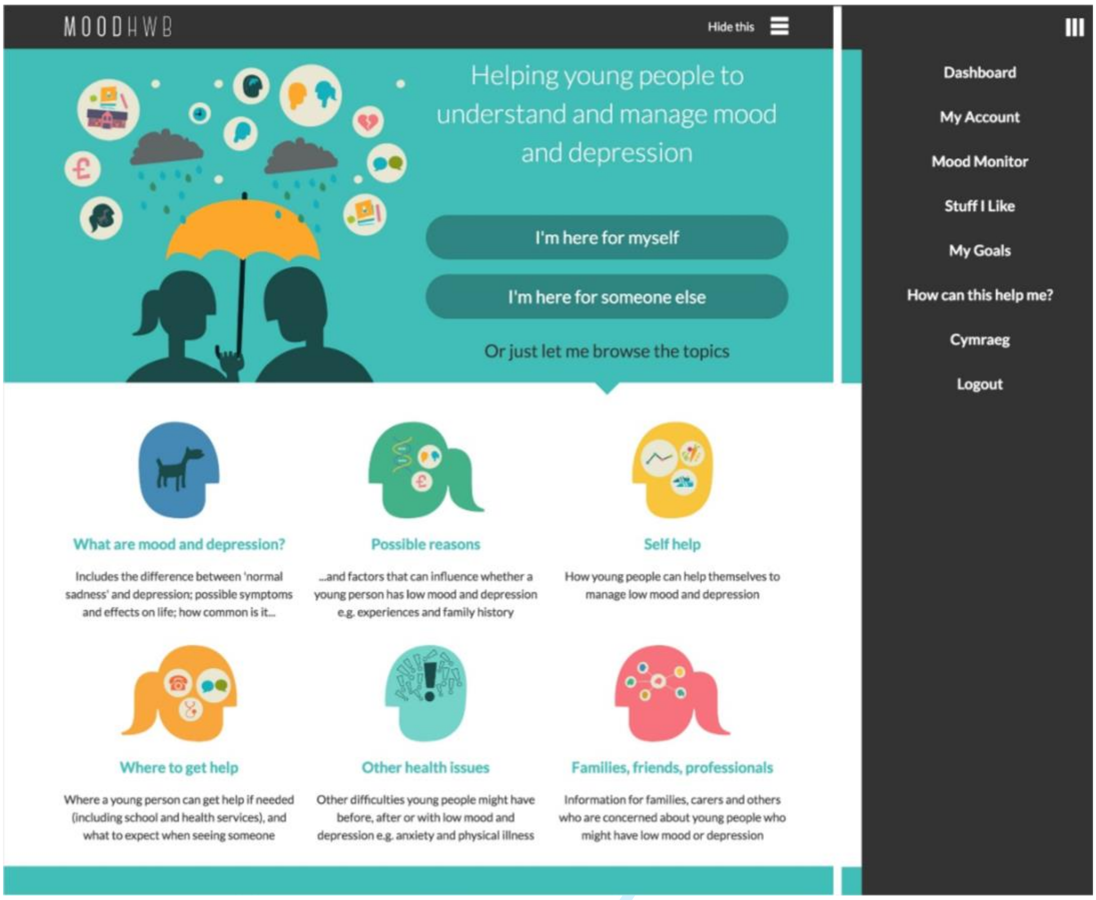

Figure 1:

MoodHwb (v1) welcome screen (main image/left) and open menu (right)

(from Bevan Jones et al. 2020a)

view only

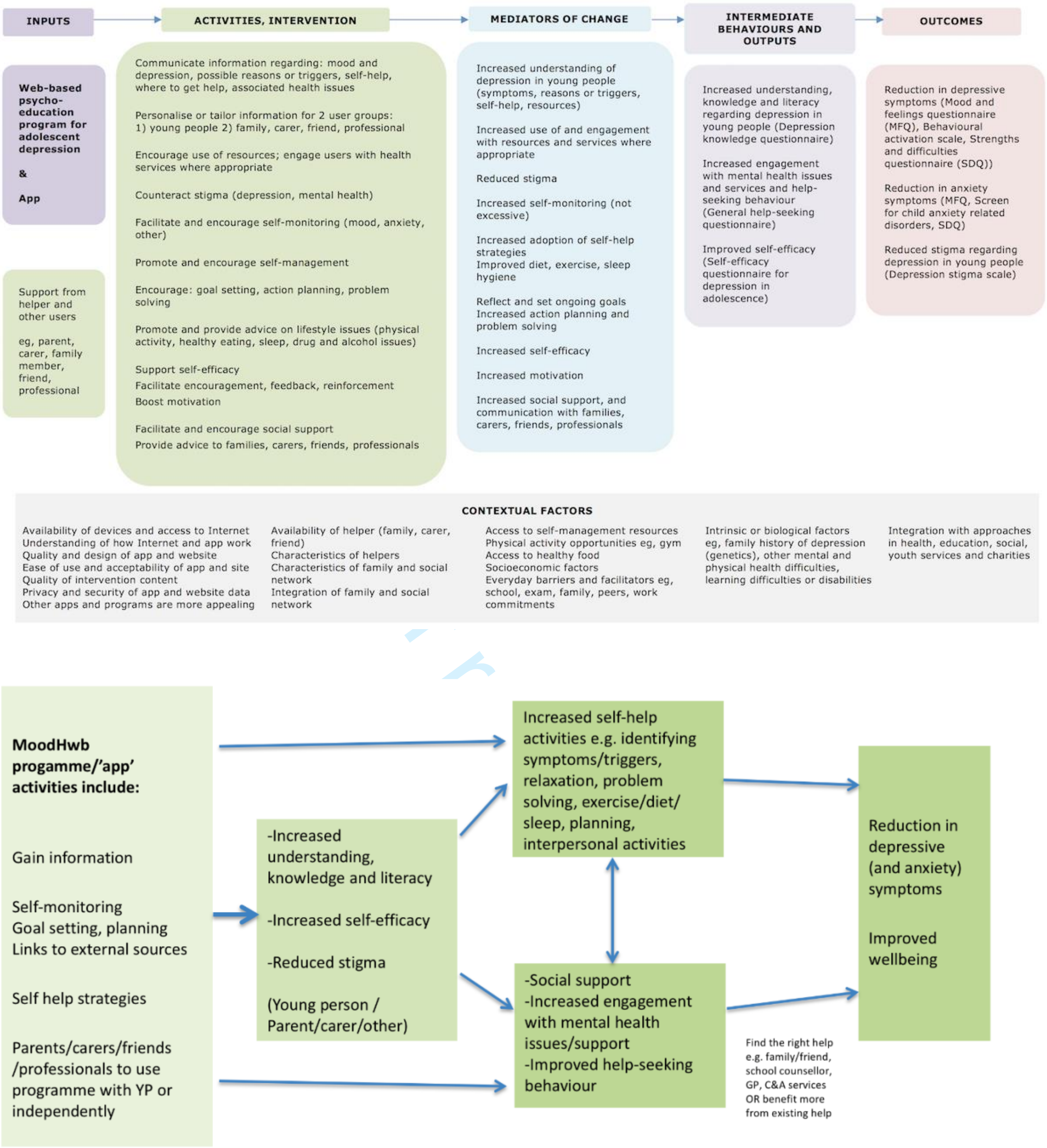

Figure 2:

Logic model for MoodHwb (above, from Bevan Jones et al 2018b), including potential pathways (below)

(applies primarily to the young person using the programme, although other users (parent/carer/other) might also benefit from the programme through e.g. increased depression literacy/knowledge or reduced stigma)

Figure 3: Participant flow diagram

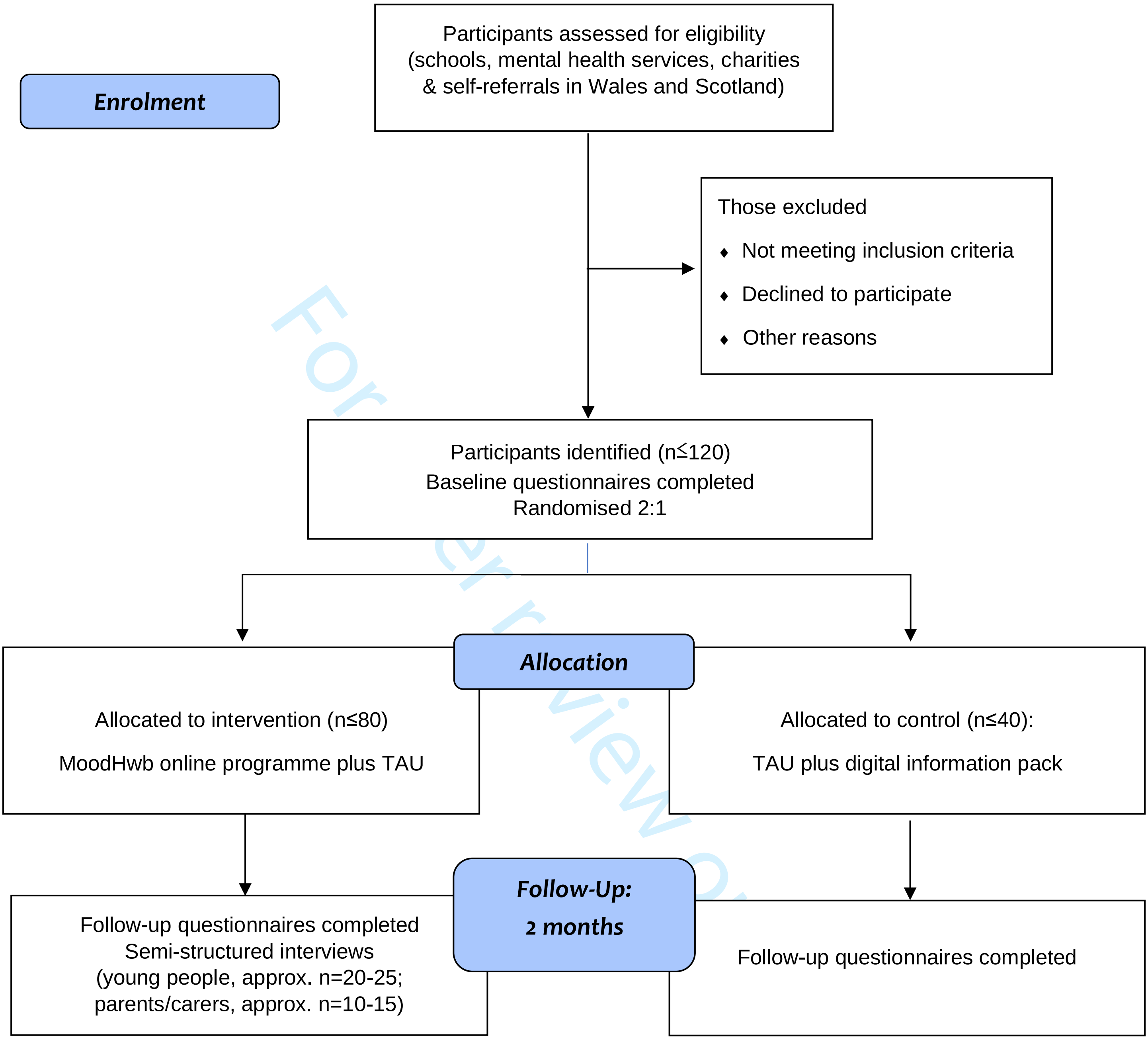

NB: Web/app usage data of those in the intervention arm will collected for 6 months after baseline.

For peer review only - http://bmjopen.bmj.com/site/about/guidelines.xhtml

Proposed progression criteria – 'Digital support for young people with their mood and wellbeing' study

**CRITERIA**	**INDICATOR**<br>GREEN = very strong indication to proceed<br>AMBER = medium indication to proceed. Discuss with Trial Steering Committee (TSC) and proceed with identified plan.<br>RED = indication of doubt as to whether to proceed. Discuss with TSC, and only proceed if other indicators are amber/green and there is a clear mitigation strategy.	**METHOD OF ASSESSMENT**
**1. Feasibility and acceptability outcomes related to the trial methods**		
1.1. Recruitment rate	Actual recruitment rate vs. Target recruitment rate:<br>Green: ≥85% of participants<br>Amber: 60-84%<br>Red: <60%	Number of eligible young people who consent to participate in the study within the first 6 months of recruitment<br>Vs. Target number (120) within this period<br><br>[Also to review % of young people who showed an interest in the study who go on to participate]
1.2. Retention rate	Retention rates:<br>Green: ≥75% of participants<br>Amber: 50-74%<br>Red: <50%	Number of young people who remained in the study at 2 months<br>Vs. Total number who consented to participate at baseline
1.3. Completeness of outcome measures	Completion of core measures at baseline and follow-up:<br>Green: ≥90% data completion	% of participants who completed the core questionnaires (including measures on depressive and anxiety symptoms, wellbeing,

For peer review only

	Amber: 70-89%<br>Red: <70%	knowledge and help-seeking)<br>% of missing data from completed core questionnaires<br><br>Both at i) baseline and ii) 2-month follow-up (i.e. based on completion by those who remained in the study)<br><br>[Also to review views/acceptability of trial methods from questionnaire, interview and focus group data]
**2. Feasibility and acceptability outcomes related to the digital programme**		
2.1. Level of usage of programme	% of participants in the intervention arm who accessed and used the programme:<br>Green: ≥80%<br>Amber: 60-79%<br>Red: <60%<br><br>Also to discuss number of times the programme has been accessed, duration of use and the sections/components accessed.	Web/app usage data from Google Analytics, based on log-ins into and use of MoodHwb by young people (and parents/carers) – up to 2 and 6 months from baseline<br><br>Questionnaire items on usage completed by young people and parents/carers at 2-month follow-up<br><br>Semi-structured interviews with young people and parents/carers at 2-month follow-up
2.2. Views or acceptability of design and content of programme	Progression to be agreed in conjunction with TSC based on data captured around design, content, and technical/accessibility aspects of MoodHwb.	Qualitative data from semi-structured interviews with young people and parents/carers after 2 months<br><br>Qualitative data from focus group with

For peer review only

	Green: If changes are needed, they are minor or have been overcome over the course of the study<br>Amber: Changes needed are significant, but feasible with further development work<br>Red: Changes needed are significant and not feasible to address	professionals<br><br>Questionnaire items on acceptability completed by young people and parents/carers at 2-month follow-up<br><br>Web/app usage data from Google Analytics (e.g. on technical/accessibility aspects), based on young person and parent/carer use of MoodHwb - up to 6 months after baseline
**3. Outcome measures for a full trial**		
3.1 Identification of primary outcomes for full trial	Discussion on the determination of primary outcome measures with TSC, including reflection on efficacy signals from candidate outcomes	Questionnaire data at 2 month follow-up<br><br>Semi-structured interviews with young people and parents/carers at 2-month follow-up<br><br>Focus group with professionals

For peer review only

STANDARD PROTOCOL ITEMS: RECOMMENDATIONS FOR INTERVENTIONAL TRIALS

SPIRIT 2013 Checklist: Recommended items to address in a clinical trial protocol and related documents*

Notes on 'Further development and feasibility randomised control trial of a digital programme for adolescent depression, MoodHwb: study protocol' (submitted to BMJ Open) in blue below. Please contact the corresponding author for further information.

Section/item	Item No	Description
**Administrative information**		
Title	1	Descriptive title identifying the study design, population, interventions, and, if applicable, trial acronym<br><br>See title page
Trial registration	2a	Trial identifier and registry name. If not yet registered, name of intended registry
	2b	All items from the World Health Organization Trial Registration Data Set<br><br>See abstract and 'Ethics and disseminations' section. Trial registration number: ISRCTN12437531
Protocol version	3	Date and version identifier<br><br>This is the first submission of the protocol for publication and is based on v7 of the trial protocol.
Funding	4	Sources and types of financial, material, and other support<br><br>See 'Funding statement'
Roles and responsibilities	5a	Names, affiliations, and roles of protocol contributors<br><br>See title page, and 'Authors' contributions'
	5b	Name and contact information for the trial sponsor<br><br>See 'Trial sponsor' section. Contact information can be requested from the corresponding author.

For peer review only - http://bmjopen.bmj.com/site/about/guidelines.xhtml

	5c	Role of study sponsor and funders, if any, in study design; collection, management, analysis, and interpretation of data; writing of the report; and the decision to submit the report for publication, including whether they will have ultimate authority over any of these activities<br><br>The sponsor and funders did not have a role in these activities
	5d	Composition, roles, and responsibilities of the coordinating centre, steering committee, endpoint adjudication committee, data management team, and other individuals or groups overseeing the trial, if applicable (see Item 21a for data monitoring committee)<br><br>See 'Trial management' section
**Introduction**		
Background and rationale	6a	Description of research question and justification for undertaking the trial, including summary of relevant studies (published and unpublished) examining benefits and harms for each intervention<br><br>See 'Background' section
	6b	Explanation for choice of comparators<br><br>See 'Trial design' and 'Intervention' sections
Objectives	7	Specific objectives or hypotheses<br><br>See 'Background' section
Trial design	8	Description of trial design including type of trial (eg, parallel group, crossover, factorial, single group), allocation ratio, and framework (eg, superiority, equivalence, noninferiority, exploratory)<br><br>See 'Trial design' section
**Methods: Participants, interventions, and outcomes**		
Study setting	9	Description of study settings (eg, community clinic, academic hospital) and list of countries where data will be collected. Reference to where list of study sites can be obtained<br><br>See 'Setting and participants' section
Eligibility criteria	10	Inclusion and exclusion criteria for participants. If applicable, eligibility criteria for study centres and individuals who will perform the interventions (eg, surgeons, psychotherapists)<br><br>See 'Setting and participants' section

For peer review only

Interventions	11a	Interventions for each group with sufficient detail to allow replication, including how and when they will be administered<br><br>See 'Intervention' section
	11b	Criteria for discontinuing or modifying allocated interventions for a given trial participant (eg, drug dose change in response to harms, participant request, or improving/worsening disease)<br><br>See 'Adverse event reporting and harms'
	11c	Strategies to improve adherence to intervention protocols, and any procedures for monitoring adherence (eg, drug tablet return, laboratory tests)<br><br>n/a
	11d	Relevant concomitant care and interventions that are permitted or prohibited during the trial<br><br>See reference to 'usual care' throughout – especially in 'Setting and participants'
Outcomes	12	Primary, secondary, and other outcomes, including the specific measurement variable (eg, systolic blood pressure), analysis metric (eg, change from baseline, final value, time to event), method of aggregation (eg, median, proportion), and time point for each outcome. Explanation of the clinical relevance of chosen efficacy and harm outcomes is strongly recommended<br><br>See 'Outcome measures' section
Participant timeline	13	Time schedule of enrolment, interventions (including any run-ins and washouts), assessments, and visits for participants. A schematic diagram is highly recommended (see Figure)<br><br>See 'Table 1' and 'Figure 3'
Sample size	14	Estimated number of participants needed to achieve study objectives and how it was determined, including clinical and statistical assumptions supporting any sample size calculations<br><br>See 'Sample size' section
Recruitment	15	Strategies for achieving adequate participant enrolment to reach target sample size<br><br>See 'Recruitment' section

For peer review only - http://bmjopen.bmj.com/site/about/guidelines.xhtml

Methods: Assignment of interventions (for controlled trials)

Allocation:

Sequence generation	16a	Method of generating the allocation sequence (eg, computer-generated random numbers), and list of any factors for stratification. To reduce predictability of a random sequence, details of any planned restriction (eg, blocking) should be provided in a separate document that is unavailable to those who enrol participants or assign interventions
Allocation concealment mechanism	16b	Mechanism of implementing the allocation sequence (eg, central telephone; sequentially numbered, opaque, sealed envelopes), describing any steps to conceal the sequence until interventions are assigned
Implementation	16c	Who will generate the allocation sequence, who will enrol participants, and who will assign participants to interventions
Blinding (masking)	17a	Who will be blinded after assignment to interventions (eg, trial participants, care providers, outcome assessors, data analysts), and how
	17b	If blinded, circumstances under which unblinding is permissible, and procedure for revealing a participant's allocated intervention during the trial
		All of the above – see 'Randomisation' section

Methods: Data collection, management, and analysis

Data collection methods	18a	Plans for assessment and collection of outcome, baseline, and other trial data, including any related processes to promote data quality (eg, duplicate measurements, training of assessors) and a description of study instruments (eg, questionnaires, laboratory tests) along with their reliability and validity, if known. Reference to where data collection forms can be found, if not in the protocol
	18b	Plans to promote participant retention and complete follow-up, including list of any outcome data to be collected for participants who discontinue or deviate from intervention protocols

For peer review only - http://bmjopen.bmj.com/site/about/guidelines.xhtml

Data management	19	Plans for data entry, coding, security, and storage, including any related processes to promote data quality (eg, double data entry; range checks for data values). Reference to where details of data management procedures can be found, if not in the protocol<br><br>All of the above – see ‘Assessment schedule’ and ‘Outcome measures’ sections
Statistical methods	20a	Statistical methods for analysing primary and secondary outcomes. Reference to where other details of the statistical analysis plan can be found, if not in the protocol
	20b	Methods for any additional analyses (eg, subgroup and adjusted analyses)
	20c	Definition of analysis population relating to protocol non-adherence (eg, as randomised analysis), and any statistical methods to handle missing data (eg, multiple imputation)<br><br>All of the above – see ‘Planned analysis’ section
**Methods: Monitoring**		
Data monitoring	21a	Composition of data monitoring committee (DMC); summary of its role and reporting structure; statement of whether it is independent from the sponsor and competing interests; and reference to where further details about its charter can be found, if not in the protocol. Alternatively, an explanation of why a DMC is not needed
	21b	Description of any interim analyses and stopping guidelines, including who will have access to these interim results and make the final decision to terminate the trial<br><br>See ‘Trial management’ section
Harms	22	Plans for collecting, assessing, reporting, and managing solicited and spontaneously reported adverse events and other unintended effects of trial interventions or trial conduct<br><br>See ‘Adverse event reporting and harms’ section
Auditing	23	Frequency and procedures for auditing trial conduct, if any, and whether the process will be independent from investigators and the sponsor<br><br>See ‘Trial management’ section
**Ethics and dissemination**		
Research ethics approval	24	Plans for seeking research ethics committee/institutional review board (REC/IRB) approval

Protocol amendments	25	Plans for communicating important protocol modifications (eg, changes to eligibility criteria, outcomes, analyses) to relevant parties (eg, investigators, REC/IRBs, trial participants, trial registries, journals, regulators)<br><br>For both – see 'Ethics and dissemination' and 'Abstract'
Consent or assent	26a	Who will obtain informed consent or assent from potential trial participants or authorised surrogates, and how (see Item 32)
	26b	Additional consent provisions for collection and use of participant data and biological specimens in ancillary studies, if applicable<br><br>All of the above - see 'Recruitment' and 'Consent' sections
Confidentiality	27	How personal information about potential and enrolled participants will be collected, shared, and maintained in order to protect confidentiality before, during, and after the trial<br><br>We will act to preserve participant confidentiality and will not disclose or reproduce any information by which participants could be identified, except where specific consent is obtained. Data will be stored in a secure manner (including on secure university servers) and will be registered in accordance with the General Data Protection Regulation 2018.
Declaration of interests	28	Financial and other competing interests for principal investigators for the overall trial and each study site<br><br>See 'Competing interests statement'
Access to data	29	Statement of who will have access to the final trial dataset, and disclosure of contractual agreements that limit such access for investigators<br><br>This can be provided, and is in the original protocol
Ancillary and post-trial care	30	Provisions, if any, for ancillary and post-trial care, and for compensation to those who suffer harm from trial participation<br><br>This can be provided
Dissemination policy	31a	Plans for investigators and sponsor to communicate trial results to participants, healthcare professionals, the public, and other relevant groups (eg, via publication, reporting in results databases, or other data sharing arrangements), including any publication restrictions<br><br>See 'Ethics and dissemination'

For peer review only

	31b	Authorship eligibility guidelines and any intended use of professional writers<br><br>We follow the BMJ Open guidelines. No professional writers were involved.
	31c	Plans, if any, for granting public access to the full protocol, participant-level dataset, and statistical code<br><br>We are submitting the protocol for publication.
**Appendices**		
Informed consent materials	32	Model consent form and other related documentation given to participants and authorised surrogates<br><br>See examples of consent forms as supplementary documents.
Biological specimens	33	Plans for collection, laboratory evaluation, and storage of biological specimens for genetic or molecular analysis in the current trial and for future use in ancillary studies, if applicable<br><br>N/A

*It is strongly recommended that this checklist be read in conjunction with the SPIRIT 2013 Explanation & Elaboration for important clarification on the items. Amendments to the protocol should be tracked and dated. The SPIRIT checklist is copyrighted by the SPIRIT Group under the Creative Commons "Attribution-NonCommercial-NoDerivs 3.0 Unported" license.

For peer review only
